# DNA hypermethylation contributes to colorectal cancer metastasis by regulating the binding of CEBPB and TFCP2 to the *CPEB1* promoter

**DOI:** 10.1186/s13148-021-01071-z

**Published:** 2021-04-23

**Authors:** Keke Shao, Weilin Pu, Jianfeng Zhang, Shicheng Guo, Fei Qian, Ingrid Glurich, Qing Jin, Yanyun Ma, Shaoqing Ju, Zhao Zhang, Weifeng Ding

**Affiliations:** 1 Department of Laboratory Medicine, the First People’s Hospital of Yancheng City/Affiliated Hospital 4 of Nantong University, Yancheng, Jiangsu Province China; 2 Department of Laboratory Medicine, Affiliated Hospital of Nantong University, Nantong, Jiangsu Province China; 3 State Key Laboratory of Genetic Engineering, Collaborative Innovation Center for Genetics and Development, School of Life Sciences, Fudan University, Shanghai, China; 4 Department of Gastroenterology, Affiliated Hospital of Nantong University, Nantong, Jiangsu Province China; 5 Department of Medical Genetics, School of Medicine and Public Health, University of Wisconsin-Madison, Madison, WI USA; 6 Center for Precision Medicine Research, Marshfield Clinic Research Institute, Marshfield, WI USA; 7 Department of Gastrointestinal Surgery, Affiliated Hospital of Nantong University, Nantong, Jiangsu Province China; 8 Office of Research Support Services, Marshfield Clinic Research Institute, Marshfield, WI USA; 9 Department of Pathology, Affiliated Hospital of Nantong University, Nantong, Jiangsu Province China; 10 Six Industrial Research Institute, Fudan University, Shanghai, China; 11 Human Phenome Institute, Fudan University, Shanghai, China; 12 Department of Biochemistry and Molecular Biology, McGovern Medical School at The University of Texas Health Science Center at Houston, Houston, TX 77030 USA

**Keywords:** *CPEB1*, Methylation, Colorectal cancer, CEBPB, TFCP2, Metastasis, Chromatin accessibility

## Abstract

**Background:**

Aberrant DNA methylation has been firmly established as a factor contributing to the pathogenesis of colorectal cancer (CRC) via its capacity to silence tumour suppressor genes. However, the methylation status of multiple tumour suppressor genes and their roles in promoting CRC metastasis are not well characterised.

**Methods:**

We explored the methylation and expression profiles of *CPEB1* (the gene encoding cytoplasmic polyadenylation element-binding protein 1), a candidate CRC tumour suppressor gene, using The Cancer Genome Atlas (TCGA) database and validated these results in both CRC cell lines and cells from Han Chinese CRC patients (*n* = 104). The functional role of *CPEB1* in CRC was examined in experiments performed in vitro and in vivo. A candidate transcription factor capable of regulating *CPEB1* expression was predicted in silico and validated by luciferase reporter, DNA pull-down, and electrophoretic mobility shift assays.

**Results:**

Hypermethylation and decreased expression of *CPEB1* in CRC tumour tissues were revealed by TCGA database. We also identified a significant inverse correlation (Pearson’s *R* = − 0.43, *P* < 0.001) between promoter methylation and *CPEB1* expression. We validated these results in CRC samples and two CRC cell lines. We also demonstrated that up-regulation of *CPEB1* resulted in significantly decreased tumour growth, migration, invasion, and tumorigenicity and promoted tumour cell apoptosis both in vitro and in vivo. We identified the transcription factors CCAAT enhancer-binding protein beta (CEBPB) and transcription factor CP2 (TFCP2) as critical regulators of *CPEB1* expression. Hypermethylation of the *CPEB1* promoter resulted in a simultaneous increase in the capacity for TFCP2 binding and a decreased likelihood of CEBPB binding, both of which led to diminished expression of *CPEB1*.

**Conclusions:**

Our results identified a novel tumour-suppressive role of *CPEB1* in CRC and found that hypermethylation of the *CPEB1* promoter may lead to diminished expression due to decreased chromatin accessibility and transcription factor binding. Collectively, these results suggest a potential role for *CPEB1* in the diagnosis and treatment of CRC.

**Supplementary Information:**

The online version contains supplementary material available at 10.1186/s13148-021-01071-z.

## Introduction

Colorectal cancer (CRC) is the third most common cancer diagnosed and the second leading cause of cancer-related deaths in the USA [1, 2]. Recent reports document similar epidemiology for CRC in China [3]. Malignant transformation and especially distant metastasis, particularly those found in the lung and liver, are amongst the major causes of CRC-associated mortality. Invasive metastatic disease in the liver or other organs occurs in 30–60% of patients diagnosed with CRC. The five-year survival rate for CRC patients with hepatic metastasis has been estimated at 12–30%, although it can be as low as 5% in patients who do not undergo treatment [4, 5]. There are very few effective therapeutic strategies that can be used to treat CRC patients with distant metastases. This is primarily due to a lack of molecular drug targets and/or any clear understanding of molecular mechanisms underlying the emergence of its invasive potential and the establishment of metastatic disease.

DNA methylation is a form of epigenetic modification that has been identified as critical for numerous physiological processes, including embryonic development [6, 7], histone modification [8], and gene imprinting [9]. In general, methylation at a gene promoter serves to repress its expression by inhibiting transcription factor (TF) binding. By contrast, methylation within the body of a gene may enhance its expression [8, 10, 11]. Widespread alterations in DNA methylation patterns have been identified and are considered to be amongst the driving forces promoting growth and metastasis of multiple tumour types. Specifically, DNA hypermethylation can silence tumour suppressor genes (TSGs), thereby leading to tumorigenicity, development, and metastasis [12]. Although altered DNA methylation patterns of some CRC-associated TSGs are already well characterised, the status of others remains largely unknown [13–15]. An extensive study of these TSGs might provide insight into new drug targets to be used for treatment or early diagnosis of CRC.

Cytoplasmic polyadenylation element-binding protein 1 (*CPEB1*) encodes a sequence-specific RNA-binding protein that regulates mRNA polyadenylation and translation [16–18] and has been linked to cancer progression and metastasis [19, 20]. CPEB1 could target the 3′-UTR of *SIRT1* mRNA (encoding Sirtuin 1), thereby controlling the length of the poly(A) tail and suppressing its capacity to mediate cancer cell stemness both in vitro and in vivo [21, 22]. Numerous reports have documented a role for *CPEB1* in the development of gastric cancer [18], breast cancer [19], glioma [20, 23], and hepatocellular carcinoma [22]. However, the role of *CPEB1* and its methylation status in CRC remains unclear. In this study, we screened The Cancer Genome Atlas (TCGA) database to determine the methylation status and expression levels of *CPEB1* in CRC. We validated these findings using targeted bisulfite sequencing to evaluate *CPEB1* expression in tumour and para-tumour tissues collected from Han Chinese CRC patients. Furthermore, to confirm the role of *CPEB1* in the development of CRC, we performed gain- and loss-of-function experiments to determine the effects of *CPEB1* expression on cell proliferation, migration, invasion, and apoptosis both in vitro and in vivo. Finally, we verified promoter hypermethylation as the underlying cause of *CPEB1* down-regulation using dual-luciferase reporter, chromatin immunoprecipitation (ChIP), DNA pull-down, mass spectrometry, and electrophoretic mobility shift (EMSA) assays.

## Results

### DNA hypermethylation of the *CPEB1* promoter in colorectal cancer

**Fig. 1:**
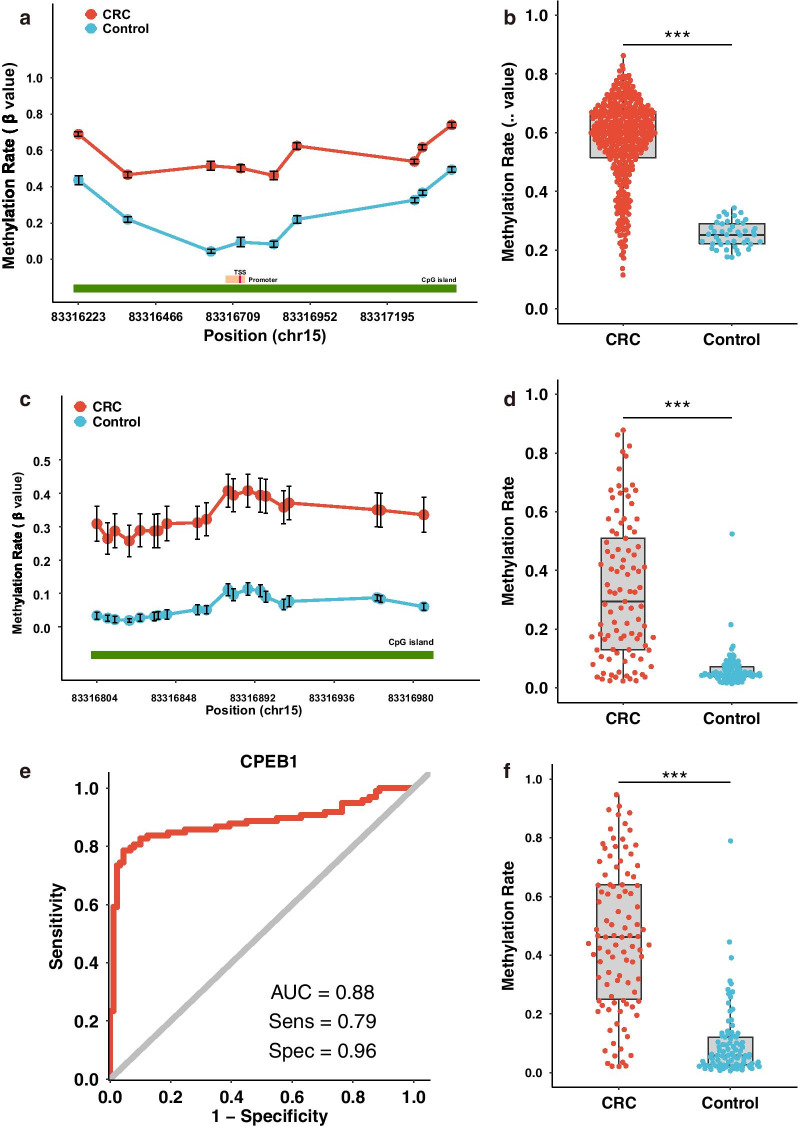
Hypermethylation of *CPEB1* in colorectal cancer. **a** The mean methylation values (*β* values) of each CpG site located at the TSS1500 of *CPEB1* in CRC tumours and para-tumour tissue identified in TCGA. The error bars represent the 95% confidence intervals (95% CIs) of the *β* values; TSS, transcription start site. **b** The average *β* value of all CpG sites located at the TSS1500 of *CPEB1* for each CRC tumour/para-tumour tissue from TCGA. **c** The mean methylation values (*β* value) of each CpG site located within our targeted region of *CPEB1* for each sample in our validation cohort (*n* = 104). The error bars represent 95% CIs of the *β* values. **d** The average *β* value of all CpG sites located within our targeted region for each CRC tumour/para-tumour tissue from the samples in our validation cohort (*n* = 104). **e** Overall ROC (receiver operating characteristics) calculated through a logistic regression model using all detected CpG sites at *CPEB1* TSS1500 included as the variables. **f** The mean methylation level of CpG sites located at the *SEPTIN9* promoter in the CRC tumours and para-tumours tissue in our validation cohort (*n* = 104). The methylation status of the *SEPTIN9* promoter served as a technical control, as methylation of this region is known to be increased in CRC compared with normal tissues

To examine the DNA methylation status of the *CPEB1* gene in CRC, we obtained the DNA methylation microarray dataset from TCGA database, including 387 CRC tumours and 45 samples of para-tumour tissue. We found that all nine of the CpG sites located within 1,500 base pairs (bps) of the transcription start site (TSS1500, including the promoter region) were significantly hypermethylated in CRC tumours compared to the para-tumour tissue (Fig. 1a, b). We validated these findings by targeted bisulfite sequencing of 104 paired samples of tumours and para-tumour tissue from Chinese Han CRC patients (Table 1). To confirm the robustness of the targeted bisulfite sequencing method, we examined the methylation status of *SEPTIN9*, a gene that has been widely reported to be hypermethylated in CRC (Fig. 1f). We found significantly higher levels of methylation of the *CPEB1* upstream region (located at chr15:83316804–83316986), which is a region close to the *CPEB1* promoter (chr15:83316688–83316747), in CRC tumours than that in the accompanying para-tumour tissues (*P* < 0.0001, Fig. 1c, d). Collectively, these results suggest that the *CPEB1* promoter region is significantly hypermethylated in CRC tissue. We also examined the potential utility of *CPEB1* hypermethylation in the diagnosis of CRC. A logistic regression model using the CpG sites of *CPEB1* promoter as variables revealed excellent sensitivity (79%) and specificity (96%) with an area under the receiving operator characteristic curve (AUC) of 0.88. Taken together, these results suggest that hypermethylation of *CPEB1* might be developed as a potential diagnostic biomarker for CRC (Fig. 1e).

**Table 1:** Characteristics of the CRC patients included in this study

Characteristics	Patient distribution
	(*n* = 104)
Age^a^	
≥ 65	58
51–64	37
≤ 50	9
Gender	
Male	71
Female	33
Subtype	
Colon	55
Rectal	49
UICC stage	
I	18
II	35
III	40
IV	11
Tumour invasion depth^b^	
T1	5
T2	21
T3	70
T4	6
Tx	2
Lymph node involvement^b^	
N0	57
N1	29
N2	11
N3	5
Nx	2
Distant metastasis^b^	
M0	93
M1	11

^a^Age stratification was done as previously described (Colorectal cancer statistics, 2020. CA Cancer J Clin, 2020)

^b^TNM stages were assessed in accordance with definitions found in the seventh edition of the TNM classification criteria

### *CPEB1* promoter hypermethylation negatively regulates gene expression

**Fig. 2:**
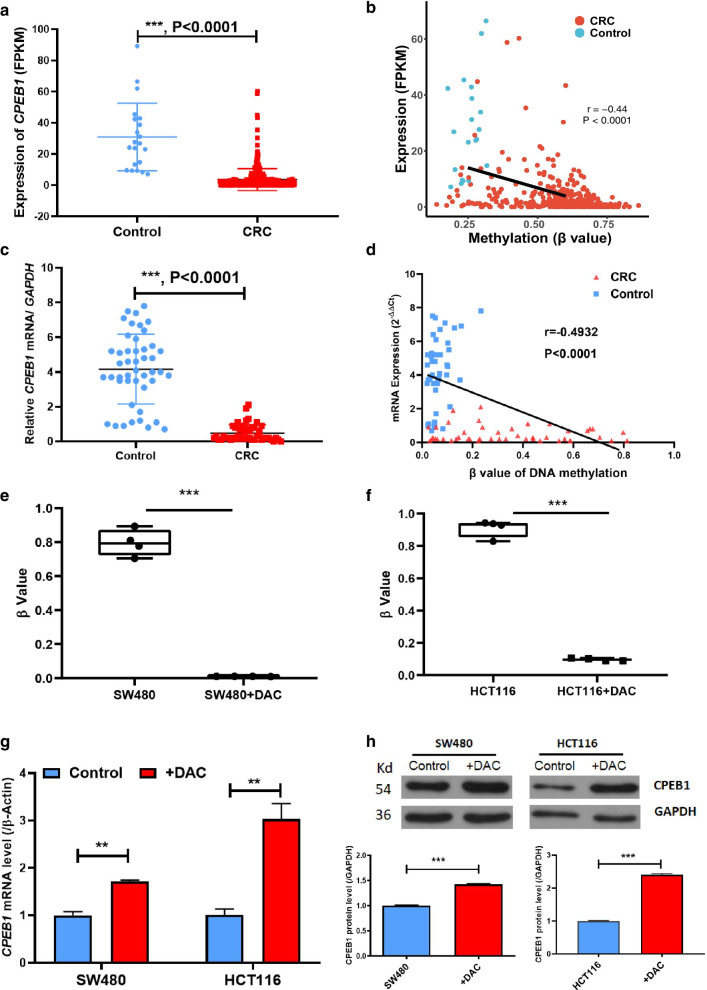
Methylation and expression levels of *CPEB1* are negatively correlated with one another in both primary CRCs and CRC cell lines. **a** Expression of *CPEB1* in CRCs and para-tumour tissues from TCGA database; FPKM, fragments per kilobase of exon model per million mapped fragments. **b** The significant negative correlation between *CPEB1* methylation and expression from the TCGA database. *CPEB1* expression is shown on the *y*-axis, whilst the average methylation level (*β* value) of the *CPEB1* promoter is shown on the *x*-axis. **c** *CPEB1* expression in CRC tumours and para-tumour tissues detected by real-time qPCR in our validation cohort. **d** The significant negative correlation between *CPEB1* methylation and expression in our validation cohort. **e**, **f** Average methylation levels of all CpG sites in the targeted region of *CPEB1* in both SW480 and HCT116 cells in the presence or absence of 5-aza-2′-deoxycytidine (DAC). Four replicate experiments were performed with each cell line. **g** Expression of *CPEB1* in SW480 and HCT116 cells in the presence or absence of DAC. **h** CPEB1 protein detected in SW480 and HCT116 cells in the presence or absence of DAC. CPEB1 protein density normalised to the GAPDH internal control is shown on the *y*-axis. Data are presented as the mean ± SD; **P < 0.01; ***P < 0.001

Multiple lines of evidence have suggested that promoter hypermethylation regulates gene expression. Findings available in TGCA dataset revealed significant *CPEB1* down-regulation in CRC tumours (Fig. 2a). Moreover, the degree of methylation of the *CPEB1* promoter inversely correlated with gene transcription (Pearson’s *R* = − 0.44, *P* < 0.0001; Fig. 2b). We validated this finding in our sample set consisting of 46 paired CRC tumour and para-tumour tissues which revealed both the down-regulation of *CPEB1* expression and the inverse correlation between transcription and *CPEB1* promoter methylation (Pearson’s *R* = − 0.49, *P* < 0.0001, Fig. 2c, d). We confirmed the inverse correlation between *CPEB1* expression and methylation of its promoter in the SW480 and HCT116 CRC cancer cell lines that were treated with the methylation inhibitor, 5-Aza-dC (5-aza-2′-deoxycytidine, DAC). We found significantly decreased *CPEB1* promoter methylation in cells treated with DAC (Fig. 2e, f) accompanied by considerable up-regulation of *CPEB1* expression (Fig. 2g). Moreover, levels of immunoreactive CPEB1 protein were also significantly higher in both cell lines after DAC treatment (Fig. 2h). Collectively, these results validated the hypermethylated status of the *CPEB1* promoter in CRC tissues and confirmed the inverse correlation between gene expression and promoter methylation of *CPEB1*.

### *CPEB1* overexpression plays a tumour-suppressing role in CRC

**Fig. 3:**
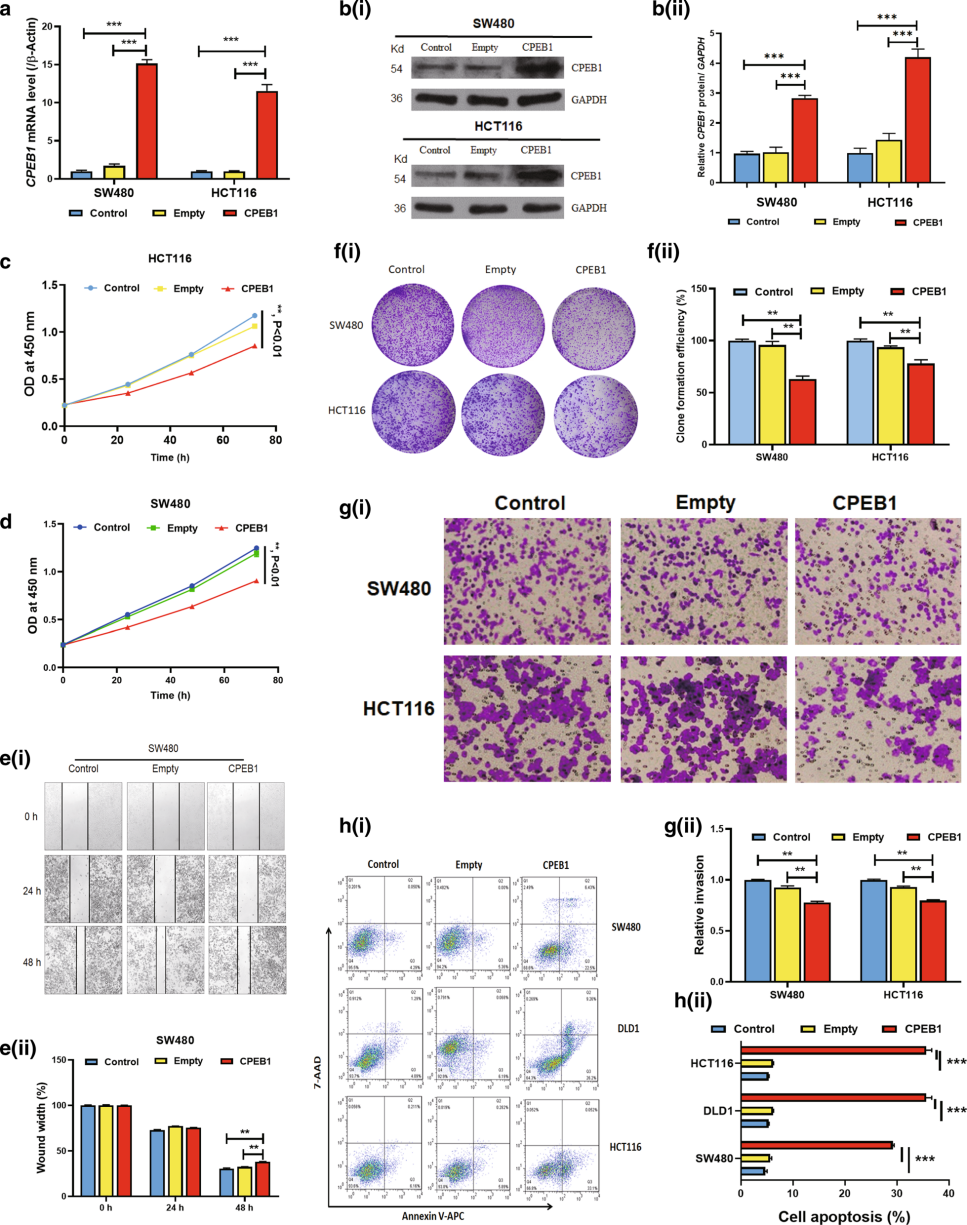
The impact of *CPEB1* overexpression on colorectal cancer cell lines in vitro. **a** Expression of *CPEB1* was evaluated in SW480 and HCT116 cells by qPCR with the expression of *ACTB* (encoding *β*-actin) used as an internal reference. Control, untransfected CRC cell lines; Empty, CRC cells transfected with the pcDNA3.1 vector alone; CPEB1, CRC cells transfected with the pcDNA3.1-CPEB1 recombinant expression vector. Levels of CBEP1/*β*-Actin mRNA presented are shown as relative *CBEP1* mRNA levels (2^−∆∆Ct^); those in the control group were normalised to 1; ***P < 0.001. **b** Immunoreactive CPEB1 was detected in SW480 and HCT116 cells by WB, with GAPDH used as an internal control. CBEP1/GAPDH are presented as relative protein intensity; values in the control group were normalised to 1. Data are presented as the mean ± SD for three independent experiments. **c**, **d** Proliferation of SW480 and HCT116 cells detected by CCK-8 assay at an OD 450 nm. **e** An in vitro scratch/wound healing assay that evaluated relative wound width over time as a measure of cellular migration. **f** CRC proliferation and growth were determined by a colony-forming assay. **g** *CPEB1* overexpression in SW480 and HCT116 cells reduced their capacity for migration and invasion in a transwell assay. h) *CPEB1* overexpression in SW480 and HCT116 cells resulted in a significant increase in the rate of cellular apoptosis. Data are presented as the mean ± SD for three independent experiments; **P < 0.01; ***P < 0.001

To determine the suppressive effects of *CPEB1* in CRC, we examined the results of *CPEB1* overexpression in the SW480 and HCT116 cell lines. *CPEB1* overexpression in pcDNA3.1-CPEB1-transfected cells (*CPEB1* group) resulted in significant increases in both *CEPB1* mRNA and CEPB1 protein (*P* < 0.0001; Fig. 3a, b) compared to cells transfected with the empty pcDNA3.1 vector alone (pcDNA3.1 group) or the untransfected control cells (control group). Cells in the *CPEB1* groups grew more slowly than the cells in the pcDNA3.1 and control group after three days in culture (Fig. 3c, d). Likewise, the proliferative capacity of the cells in the *CPEB1* group was markedly decreased compared to those in the pcDNA3.1 and control groups (Fig. 3f). The results of in vitro scratch wound healing experiments revealed that *CPEB1* overexpression in SW480 and HCT116 cells significantly inhibited cell migration (Fig. 3e and Additional file 1: Figure S4). Moreover, cells in the *CPEB1* group exhibited a diminished capacity for invasion (Fig. 3g). We also found that a significantly higher fraction of SW480, DLD-1, and HCT116 cells in the *CPEB1* group exhibited early apoptosis (Fig. 3h). Collectively, the results of these in vitro experiments suggest that expression of *CPEB1* inhibits CRC tumour cell growth, proliferation, and metastasis and promotes apoptosis.

### Increased expression of *CPEB1* in the CRC tumour xenograft model significantly inhibited metastasis

**Fig. 4:**
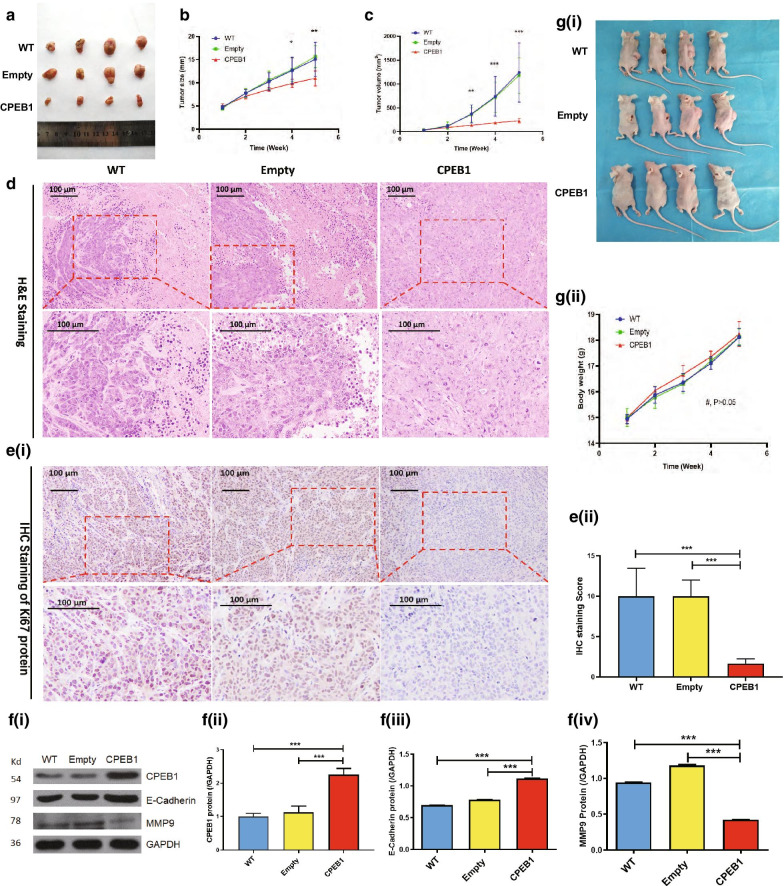
Up-regulation of *CPEB1* resulted in the inhibition of cell growth and metastasis of human colorectal cancer in vivo. **a** Xenograft tumours in mice injected with HCT116 cells that were transfected with pcDNA3.1 (Empty), pcDNA3.1-CPEB1 (CPEB1), or that were not transfected (WT) were evaluated; *n* = 4 mice per group. **b**, **c** *CPEB1* overexpression in HCT116 cells resulted in a significant decrease in tumour size (**b**) and tumour volume (**c**) in this mouse model. **d** Hematoxylin–eosin (H&E) staining of mouse tumour tissue in *CPEB1*, empty, and WT groups. **e** Immunohistochemical (IHC) staining for Ki67 verified that overexpression of *CPEB1* resulted in a reduced capacity for cell proliferation. **f** WB revealed an increased level of E-cadherin and a decreased level of MMP-9 in the mice CRC tumours derived from cell lines transfected with the *CPEB1-*containing recombinant plasmid. **g** Body weights of injected mice were recorded throughout; WT, mice injected with untransfected CRC cells; Empty, mice injected with CRC cells that were transfected with the pcDNA3.1 empty vector; CPEB1, mice transfected with the pcDNA3.1-CPEB1 recombination plasmid vector; ^#^P > 0.05 indicates no statistical significance between results from WT, Empty, and CPEB1 groups; *P < 0.05; **P < 0.01; ***P < 0.001

To determine whether *CPEB1* functions as a CRC TSG in vivo, we established a xenograft mouse model. HCT116 cells transfected with either the plasmid pcDNA3.1-CPEB1 (*CPEB1* group) or pcDNA3.1 (pcDNA3.1 group) were inoculated into BALB/c nude mice. The mice were sacrificed 36 days later and tumour volumes and wet weights were recorded. The tumours of the mice in the *CPEB1* group were visibly smaller and of significantly smaller volume than those in the pcDNA3.1 and control groups (Fig. 4a, b, c). However, we detected no significant difference in total mouse body weight amongst the three groups at any time point during this study (Fig. 4g). We observed no abnormalities with respect to daily food and water consumption or any other adverse effects, such as altered behaviours and haematuria.

Tumour tissue was subjected to H&E staining. Our results from this analysis suggested that higher expression of *CPEB1* resulted in significantly diminished carcinogenesis in this in vivo model of CRC (Fig. 4d). To demonstrate the anti-proliferative and anti-metastatic role of *CPEB1 *in vivo, we examined the levels of immunoreactive Ki67, matrix metallopeptidase-9 (MMP-9), and E-cadherin in mouse tumour tissues by immunohistochemistry (IHC) and Western blotting (WB). We found that levels of immunoreactive Ki67 and MMP-9 proteins were diminished in tumours isolated from the *CPEB1* group. By contrast, the levels of E-cadherin, a signal biomarker for epithelial–mesenchymal transition, were significantly increased in the *CPEB1* group (Fig. 4e, f). Taken together, the results from the xenograft model result strongly support a role for *CPEB1* as a TSG with the capacity to prevent CRC tumorigenesis and metastasis.

### Methylation of the CEBPB-binding site inhibits the transcription of *CPEB1*

**Fig. 5:**
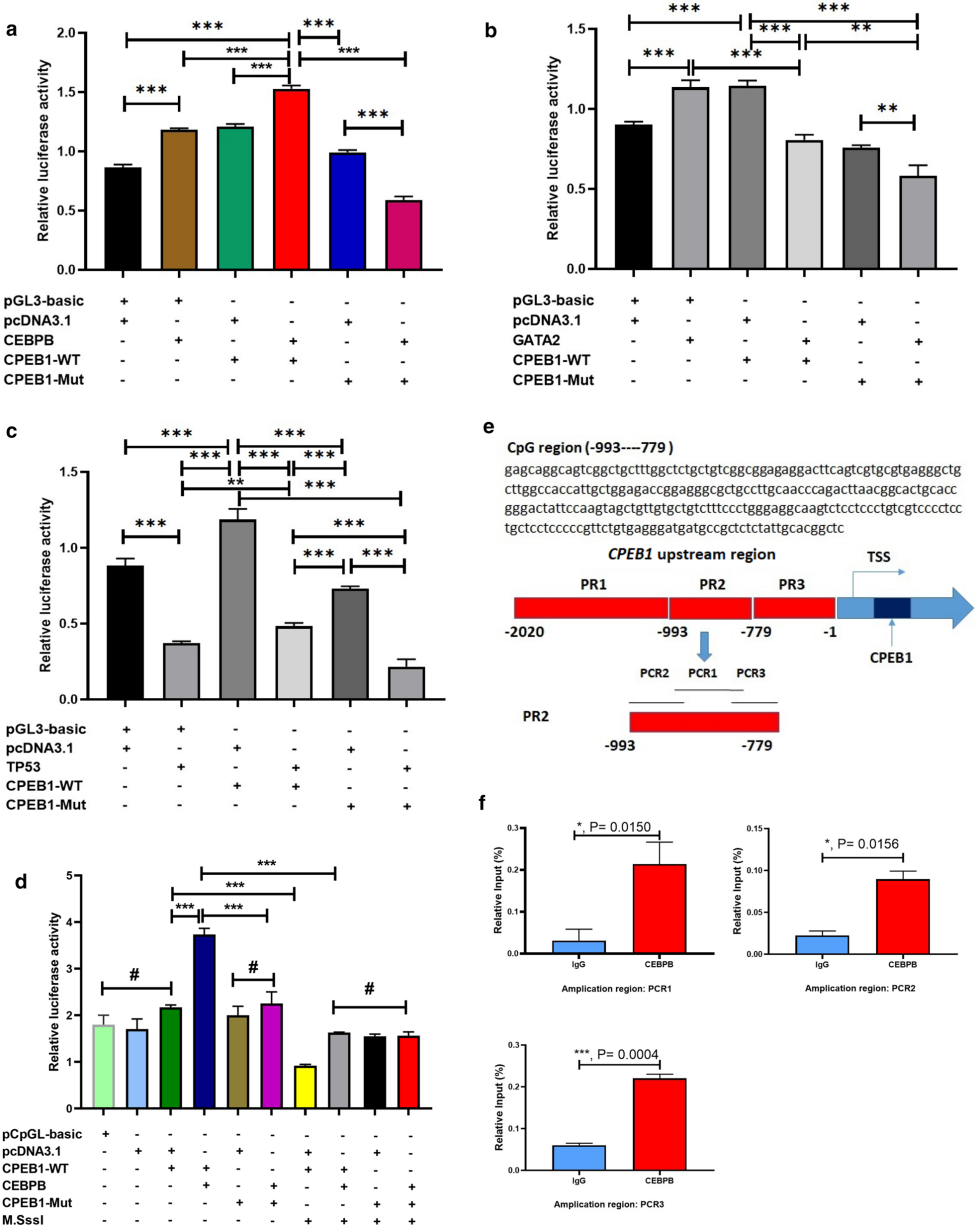
In silico predictions and dual-luciferase reporter gene assays identified CEBPB as a key transcription factor (TF) binding to the upstream region of *CPEB1.* **a**–**c** Dual-luciferase reporter assays revealed that CEBPB binds at the *CPEB1* upstream region in HCT116 cells and functions as a key TF. CEBPB, cells transfected with the pcDNA3.1-CEBPB vector; CPEB1-WT, cells transfected with the pGL3-CPEB1 vector that contains the wild-type *CPEB1* promoter region; CPEB1-Mut, cells transfected with the pGL3-CPEB1 vector that contains a mutated version of the *CPEB1* promoter region; GATA2, cells transfected with a pcDNA3.1-GATA2 vector; TP53, cells transfected with a pcDNA3.1-TP53 vector; **P < 0.01; ***P < 0.001. **d** Dual-luciferase reporter assays revealed that hypermethylation of the CEBPB-binding region resulted in diminished activation of *CPEB1* by CEBPB. CPEB1-WT and CPEB1-Mut represent the wild-type and mutated forms of the *CPEB1* promoter, respectively, inserted into the (CpG-free) pCpGL vector; M.Sssl, CpG methyltransferase, an enzyme that can methylate the CpGs in sequences inserted into the pCpGL vector; **P < 0.01; ***P < 0.001, ^#^*P* > 0.05. **e** The flowchart documenting the steps involved in the ChIP-PCR assay. The upstream region of *CPEB1* (approximately 2020 bp) was divided into PR1, PR2, and PR3. PR2, which was the predicted core TF-binding region (− 993 to − 779) was further divided into PCR1, PCR2, and PCR3 as shown; TSS, transcription start site. **f** ChIP-PCR assay validated CEBPB binding to the *CPEB1* core TF-binding region (− 993 to − 779) in HCT116 cells. *CPEB1* expression levels were determined using the DNA pulled-down in the ChIP assay with various antibodies. Relative input (%) = *CPEB1* expression in the DNA pulled-down with anti-CEBPB or anti-IgG (control)/Input. ***P < 0.001

Altered DNA methylation at the *CPEB1* promoter region could regulate gene expression by inhibiting TF binding. However, it was not clear which TFs were crucial for the regulation of *CPEB1* expression in CRC. We identified three candidate TFs using PROMO and JASPAR software, including CCAAT Enhancer-Binding Protein Beta (*CEBPB*), GATA-binding factor 2 (*GATA2*), and tumour protein P53 (*TP53*) (Additional file 1: Figure S1). We then performed dual-luciferase reporter assays to determine which of these TFs could regulate the expression of *CPEB1*. As shown in Fig. 5a, the transcriptional activity in cells transfected with both CPEB1-WT and pGL3-basic was significantly increased in response to CEBPB. Although the luciferase activities detected in both of these groups increased in response to CEBPB, the relative activity detected in the CEBPB + CPEB1-WT group was significantly higher than that detected in the CEBPB + pGL3-basic group. These results suggested that CEBPB was capable of significant activation of *CPEB1* expression. By contrast, the introduction of mutations into the TF-binding region (CPEB1-Mut) substantially reduced the transcriptional activation of CPEB1 by CEBPB. These results indicated that the identified TF-binding region is essential for CEBPB-mediated activation of *CPEB1* expression. In contrast to our findings for CEBPB, neither GATA2 nor TP53 could enhance the transcriptional activity of *CPEB1* (Fig. 5b, c). Collectively, our results suggested that CEBPB might be critical TF regulating *CPEB1* expression in CRC cells.

We then performed dual-luciferase reporter assays to determine whether hypermethylation at the CEBPB-binding site would have an impact on the expression of *CPEB1*. For these experiments, we generated luciferase reporter constructs that included either non-methylated or hypermethylated fragments of the upstream region of *CPEB1* that included the CEBPB-binding site. We found that hypermethylation of the CEBPB-binding site (i.e. CPEB1 WT + CEBPB + M.SssI) resulted in a significant decrease in luciferase activity. These results suggest that hypermethylation of the CEBPB-binding site could inhibit *CPEB1* expression via interference with the recruitment of CEBPB to the *CPEB1* promoter region (Fig. 5d). To evaluate this possibility, we performed a ChIP assay that demonstrated direct CEBPB binding to its cognate site in the upstream fragment of *CPEB1* using the cultured cells transfected with pcDNA3.1-CEBPB vector (Fig. 5e). Specifically, we found that the -993 to -779 upstream region of the *CPEB1* promoter region bound CEBPB more effectively than the IgG control. These results suggest that CEBPB was capable of direct binding to the -993 to -779 region of the *CPEB1* promoter and thus could regulate *CPEB1* transcription (Fig. 5f).

### Hypermethylation of the *CPEB1* promoter results in increased binding of TFCP2 instead of CEBPB

**Fig. 6:**
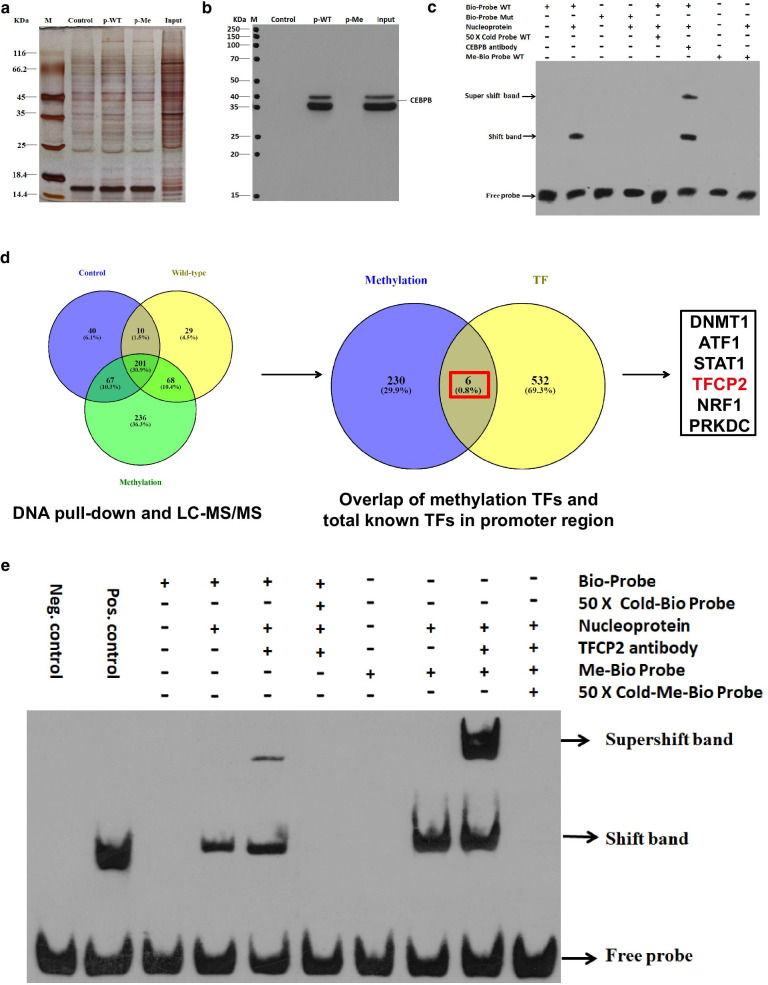
TFCP2 binds to the core TF-binding region of *CPEB1* when it is hypermethylated. **a** Silver staining of the nucleoproteins identified in DNA pull-down assay under various conditions. p-WT, the non-methylated *CPEB1* promoter; p-Me, the hypermethylated *CPEB1* promoter; control, magnetic beads without probes; Input, total nucleoproteins extracted from HCT116 cells; M, protein molecular mass marker. **b** WB detecting immunoreactive CEBPB in the nucleoprotein fraction after DNA pull-down with the anti-CEBPB antibody. The molecular mass of CEBPB is approximately 35 kDa. **c** EMSA revealed that CEBPB protein was unable to bind to its target sequence in the hypermethylated TF-binding region of *CPEB1*; 50 × cold probe WT, 50-fold concentration of the unlabelled wild-type *CPEB1* promoter which was served as the competitor probe; Bio-Probe WT, a biotin-labelled wild-type probe of *CPEB1* upstream region; Bio-Probe Mut, a biotin-labelled mutant probe of *CPEB1* upstream region; Nucleoprotein, nucleoprotein extracted from HCT116 cells; Me-Bio Probe WT, a biotin-labelled hypermethylated probe of *CPEB1* upstream region. **d** TFCP2 may be a candidate methylation reader at the upstream region of *CPEB1*; Methylation, the hypermethylated *CPEB1* upstream region probe; Wild-type, the wild-type *CPEB1* upstream region probe; Control, a probe with a scrambled sequence of *CPEB1* upstream region; TF, the TFs capable of binding to the *CPEB1* upstream as determined by ChIP-Seq. **e** Competitive EMSA to confirm TFCP2 as a methylation reader for *CPEB1*. Bio-probe, a biotin-labelled wild-type *CPEB1* upstream region probe; Me-Bio Probe, a biotin-labelled hypermethylated *CPEB1* upstream region probe; 50 × Cold Probe, 50-fold concentration of the unlabelled wild-type *CPEB1* upstream region probe that served as a competitor of the Bio-probe; 50 × Cold Me-Probe, 50-fold concentration the unlabelled hypermethylated *CPEB1* upstream region probe that served as a competitor of the Me-Bio Probe

We then performed DNA pull-down/mass spectrometry (MS) experiments to explore TF binding to the hypermethylated *CPEB1* promoter. Consistent with our previous results, we confirmed that hypermethylation of the TF-binding site resulted in markedly reduced binding of CEBPB (Fig. 6a, b). We also found that the notable supershifted band on electromobility shift assay (EMSA) representing CEBPB interactions with the wild-type *CPEB1* upstream region probe could not be detected in experiments performed with either mutant or hypermethylated probes (Fig. 6c). These results add strength to our conclusion that CEBPB can bind to the *CPEB1* upstream region directly and that hypermethylation of the CEBPB-binding region results in reduced binding and diminished expression of *CPEB1*.

Finally, we analysed the proteins in the complexes that were pulled down by the methylated *CPEB1* probe using MS. We identified 236 candidate binding proteins in this experiment (Additional file 1: Table S6). We then selected genes that were also included in the list of candidate binding proteins and the predicted TFs capable of binding to the *CPEB1* upstream region from the ChIP-seq database. As a result of this analysis, we identified six TFs that might be capable of binding to the hypermethylated *CPEB1* promoter. Amongst them, TFCP2 was the only TF that interacts with a cognate site found in the *CPEB1* TF-binding region; these results suggested that TFCP2 might be capable of substituting for CEBPB and binding to the methylated upstream region of *CPEB1* (Fig. 6d). We performed an EMSA assay to determine whether TFCP2 was capable of binding to the methylated form of the *CPEB1* upstream region. The EMSA assay revealed that hypermethylation of the *CPEB1* TF-binding region enhanced the binding interaction between TFCP2 and *CPEB1* (Fig. 6e). Collectively, our findings suggest that TFCP2 may bind to the methylated TF-binding region of *CPEB1* and thereby repress *CPEB1* transcription.

## Materials and methods

### Human tissues, cell lines, transfection, and drug treatment

We collected the pairs of tumour tissue specimens and their corresponding para-tumour tissues (the corresponding adjacent normal tissue resected at least 5 cm distant from the tumour tissue) from Han Chinese patients diagnosed with CRC (*n* = 104). The samples were obtained from the Department of General Surgery in Affiliated Hospital of Nantong University. Tumours were classified according to Tumour Node Metastasis (TNM)/Union for International Cancer Control (UICC) criteria following histopathological examination. The SW480, DLD1, and HCT116 cell lines were purchased from the Cell Bank of the Type Culture Collection of Chinese Academy of Sciences (Shanghai, China). The SW480 cells were cultured in Dulbecco’s modified Eagle’s medium (DMEM) with high glucose (Hyclone, USA). The HCT116 cells were grown in McCoy’s 5A medium (Thermo Fisher Scientific, USA), supplemented with 10% fetal bovine serum (FBS; Gibco, USA) at 37 °C in 5% CO_2_. The full-length cDNA of *CPEB1* (NM_030594.4) was cloned in the pcDNA3.1-EGFP (enhanced green fluorescent protein) vector to create the pcDNA3.1-CPEB1 overexpression vector. This vector was transfected into cells using Lipofectamine 2000 (Invitrogen, USA). 5-Aza-2′-deoxycytidine (DAC; Sigma-Aldrich, USA) was used for DNA demethylation as previously described [24].

### DNA methylation status of *CPEB1* in TCGA database and our validation cohort

Publicly available high-throughput DNA methylation microarray data (Illumina Human Methylation 450 K) of 387 CRC tumours and 45 samples of para-tumour tissue were obtained from the TCGA database (level-3). We identified nine candidate CpG sites within the *CPEB1* promoter region. The extent of methylation (*β* value) at each CpG site was calculated based on the hypermethylated (*M*) and non-methylated (*U*) intensities as shown:

$$\beta \;{\text{value}} = \frac{{\max \left( {M,0} \right)}}{{\max \left( {M,0} \right) + \max \left( {U,0} \right).}}$$

The *β* values calculated for CRC tumours and para-tumours were compared at each candidate CpG site. The mean methylation level of the CpG sites for each tissue sample was calculated as overall *CPEB1* methylation. We then compared mean methylation levels of individual CRC tumours and para-tumours.

To validate these findings, we extracted DNA samples from both tumour and paired para-tumour tissue which was subjected to bisulfite conversion. After careful evaluation of the CG percentage, poly(T), and single-nucleotide polymorphisms (SNPs) in the potentially differentially methylated regions (DMRs), we designed primers to detect the methylation of *CPEB1* upstream region using next-generation sequencing (NGS). The amplified DNA fragments were sequenced by Illumina Hiseq 2000 (Illumina, San Diego, CA, USA). To verify the robustness of our methodology, we also examined the methylation status of the *SEPTIN9*, a gene known to be hypermethylated in CRC tissues (Additional file 1: Table S1). The detailed protocol used for targeted bisulfite sequencing was described previously [24]. We applied BSseeker2 to map reads and to identify methylated sites. Samples with high missing rates (> 30%) and CpG sites with high missing rates (> 20%) were removed from the dataset. The methylation rate (*β* value) for each CpG site was calculated according to the number of reads (C or T) detected at each CpG site.

$${\text{Methylation}}\;{\text{rate}} = \frac{n\left( C \right)}{{n\left( C \right) + n\left( T \right)}}.$$

Similarly, the mean methylation rate at each of these CpG sites in each sample was considered as the overall methylation of *CPEB1*. Overall methylation of *CPEB1* was compared in both CRC tumours and their associated para-tumour tissue. The methylation rate (*β* value) for each CpG site and for each sample determined in the validation stage is shown in Additional file 2: Table S7.

### RNA extraction and real-time quantitative PCR

Total RNA from CRC tissues and cell lines was isolated with TRIzol (Thermo Fisher Scientific, USA) following the manufacturer’s instructions. First-strand complementary DNA (cDNA) was synthesised from 1 μg total RNA using an M-MLV Reverse Transcriptase Kit (ELK Biotech., China). Real-time quantitative PCR was performed on an Applied Biosystems 7500 real-time PCR system (ABI, USA) with SYBR green I dye (ELK Biotech., China). The expression levels of *ACTB* (encoding *β*-actin) and *GAPDH* served as internal references.

### Cell proliferation assay

SW480 and HCT116 cells transfected with pcDNA3.1-CPEB1 and pcDNA3.1-EGFP vector-only control were incubated for 0, 24, 48, and 72 h in 96-well plates at 4,000 cells/well. Cell proliferation was detected by adding 10 µL CCK-8 solution (Biolite Biotech Co., China) to each well followed by the optical density (OD 450 nm) measurements 3 h later using a microplate reader (Thermo Fisher Scientific).

### Cell clone formation assay for cell proliferation and growth

Cells were seeded into 24-well ultra-low attachment round-bottom plates (Excel Scientific, USA) at a density of 100 cells per well in cell culture medium together with pcDNA3.1-CPEB1 and pcDNA3.1-EGFP vectors, respectively, and cultured for 14 days. Wells were then washed twice with phosphate-buffered saline (PBS), fixed for 15 min with 500 μL 4% paraformaldehyde, and stained with Giemsa dye for 10 min. The number of violet-stained cell spheres (nb: only cell spheres with > 10 cells were included) was determined in each well. The efficiency of cell colony formation was calculated as:

$${\text{Cell}}\;{\text{colony}}\;{\text{formation}}\;{\text{efficiency}}\left( \% \right) = \frac{{{\text{The}}\;{\text{number}}\;{\text{of}}\;{\text{cell}}\;{\text{spheres}}\;{\text{in}}\;{\text{experimental}}\;{\text{groups}}}}{{{\text{The}}\;{\text{number}}\;{\text{of}}\;{\text{cell}}\;{\text{spheres}}\;{\text{in}}\;{\text{control}}\;{\text{groups}}}} \times 100\% .$$

### Transwell assays to examine cell metastasis and invasion

A cell invasion assay was performed using an invasion chamber (Corning, USA) in a 24-well tissue culture plate with 12-cell culture inserts. Suspensions of cells (10^5^ cells/mL) that were transfected with pcDNA3.1-CPEB1 or pcDNA3.1-EGFP or mock-transfected with medium alone were added to the interior of the inserts in 200 μL serum-free medium. Five hundred μL of medium containing 20% FBS was added to the lower chamber followed by incubation at 37 °C in 5% CO_2_ for 24 h. Invasive cells detected on the lower surface of the membrane by staining with 0.1% crystal violet for 10 min were photographed. The number of invasive cells was calculated from images photographed at high magnification (400-fold). Relative invasion was defined as follows:

$${\text{Relative}}\;{\text{invasio}} = \frac{{{\text{The}}\;{\text{number}}\;{\text{of}}\;{\text{invasive}}\;{\text{cells}}\;{\text{in}}\;{\text{experimental}}\;{\text{groups}}}}{{{\text{The}}\;{\text{number}}\;{\text{of}}\;{\text{invasive}}\;{\text{cells}}\;{\text{in}}\;{\text{control}}\;{\text{groups}}}}.$$

### Wound healing assay to evaluate cell migration

Cells were seeded into a six-well tissue culture plate at a density that will permit them to reach 70%–80% confluence after 24 h of growth. The monolayer was scratched gently and slowly across the centre of the well with a 200-µL pipette tip. After scratching, the well was gently washed twice with tissue culture medium to remove the detached cells, and the distance between the two edges of the gap was measured (*t* = 0 h). Cells were incubated for an additional 24 and 48 h; the distances between the edges of the gap distance were assessed at these times to determine the rate of wound healing. Migration distance = gap distance at *T*_0_—gap distance at *T*_*x*_ (*T*_*x*_ = 24 h or 48 h). The gap distance was measured using ImageJ software (v1.53c). The wound width (%) was calculated as follows:

$${\text{Wound}}\;{\text{width}}\left( \% \right) = \frac{{{\text{migration}}\;{\text{distance}}\;{\text{in}}\;{\text{the}}\;{\text{experimental}}\;{\text{group}}}}{{{\text{migration}}\;{\text{distance}}\;{\text{in}}\;{\text{the}}\;{\text{control}}\;{\text{group}}}} \times 100\% .$$

### Assessment of apoptosis by flow cytometry (FCM)

Cells were removed from tissue culture plates with trypsin and washed twice in ice-cold PBS. A total of 5 × 10^5^ cells were resuspended in 500 µL of binding buffer (KeyGen, China). This was followed by the addition of 5 µL of Annexin V-APC (KeyGen) and 5 µL of the counterstain, 7-amino actinomycin D (7-AAD). The mixture was incubated at room temperature for 10 min in the dark, followed by quantitative analysis of apoptosis using a FlowSight flow cytometer (Merck, Germany).

### Dual-luciferase reporter gene assay

A dual-luciferase reporter assay system was purchased from Promega, USA. Three TFs (CEBPB, GATA, and TP53) were identified as candidate transcriptional regulators of *CPEB1*. The TF-binding regions were predicted for each factor within the upstream region of *CPEB1*. We then constructed wild-type and the mutant fragments of these regions for use in the dual-luciferase assay (Additional file 1: Table S4). The wild-type or mutant fragments of the upstream region of *CPEB1* were cloned into the pGL3-basic vector (Promega, USA) to generate CPEB1-WT and CPEB1-Mut, respectively. The sequences of all construct inserts were verified by Sanger sequencing. The full-length cDNAs of these three TFs were cloned individually into the pcDNA3.1 expression vector to generate pcDNA3.1-CEBPB, pcDNA3.1-GATA, and pcDNA3.1-TP53, respectively. HCT116 cell cultures plated on 24-well plates were transfected individually with each of the TF expression vectors (0.81–1.07 μg/μL) together with wild-type pGL3-CPEB1 (1.64–2.01 μg/μL) or mutant pGL3-CPEB1 (1.30–1.71 μg/μL) using Lipofectamine 2000. In each transfection, 0.2 μg/μL of a pRL-TK vector (Renilla luciferase vector) was used to normalise the transfection efficiency. The pGL3-basic + pcDNA3.1 group served as a negative control.

A dual-luciferase reporter assay was also performed to determine whether hypermethylation at the predicted binding site for CEBPB had an impact on the binding interaction. HCT116 cells plated on 24-well plates were transfected with 160 ng of the pcDNA3.1-CEBPB expression vector and 200 ng wild-type pCpGL-CPEB1 (i.e. the wild-type fragment of the *CPEB1* promoter region cloned into the pCpGL-basic vector), 200 ng of mutant pCpGL-CPEB1, or 200 ng of hypermethylated wild-type and mutated pCpGL-CPEB1 using Lipofectamine 2000. The pCpGL-basic vector engineered as devoid of CpG sites was a gift from Professor M. Wang, Soochow University, China. The pCpGL-CPEB1 reporter vector was hypermethylated with M.SssI (CpG) methyltransferase as per the manufacturer’s instructions (Thermo Fisher Scientific). In each transfection, 40 ng of a pRL-TK vector was used to normalise the transfection efficiency. Transfection with each of the pCpGL-basic reporter vectors together with each of the pcDNA3.1 expression vectors served as negative controls. The relative luciferase activity in each group was calculated as follows:

$${\text{Relative}}\;{\text{luciferase}}\;{\text{activity}} = \frac{{{\text{ Firefly}}\;{\text{luciferase}}\;{\text{intensity }}}}{{{\text{ Renilla}}\;{\text{luciferase}}\;{\text{intensity}}}}.$$

### ChIP-PCR assay

A chromatin immunoprecipitation (ChIP) assay was performed as previously described [24]. Briefly, HCT116 cells were transfected with pcDNA3.1-CEBPB or the control pcDNA3.1 vector. After transfection for 48 h, cells were crosslinked with 1% formaldehyde for 10 min at room temperature. Chromatin was fragmented using a sonication device (Scientz Biotechnology Company, China) to obtain DNA fragments of approximately 100–500 bp in length. Anti-CEBPB antibody (Abcam, USA) was added to form an anti-CEBPB–CEBPB protein–DNA chromatin complex. Protein A beads (Beaver Biosciences Inc., China) were used to pull down the chromatin complex. Crosslinks between DNA and proteins were destroyed by overnight incubation at 65 °C with proteinase K (Sigma-Aldrich, USA). The resulting DNA fragments were purified using the MinElute PCR Purification Kit (Aidlab Bio., China) to serve as PCR templates. We used Emboss (The European Molecular Biology Open Software Suite) software to identify the core TF-binding region of *CPEB1*. This program predicted that the region between nucleotide -993 and -779 of the *CPEB1* promoter as containing the core TF-binding region. To validate and extend this prediction, we divided the promoter region of *CPEB1* into three different parts: PR1, PR2 (containing the core TF-binding region), and PR3. Moreover, to identify the core TF-binding region by real-time quantitative PCR, we divided the core TF-binding region (PR2) into three smaller parts: PCR1, PCR2, and PCR3 (Fig. 5f). DNA purified from the ChIP experiment was used as a template to amplify the core TF-binding region of *CPEB1*. The primers used in the ChIP-PCR assay are shown in Additional file 1: Table S2. Input sample represented the fragmented, diluted, and pre-cleared chromatin DNA that was introduced into the immunoprecipitation experiment. The responses to 1% input served as the internal reference for data normalisation, and IgG was included as a negative control. Relative input (%) represented the level of *CPEB1* expression from DNA in the ChIP experiment that was precipitated with anti-CEBPB or anti-IgG.

### DNA pull-down and mass spectrometry (MS)

A DNA pull-down assay was performed as reported previously [24]. The *CPEB1* promoter region was amplified by PCR using a 5′-biotin-labelled primer (Additional file 1: Table S3); the non-methylated biotinylated double-stranded DNA was methylated with M.SssI (CpG) methyltransferase. We prepared cell lysates from HCT116 cells that were transiently transfected with the pcDNA3.1-CEBPB vector. Cell lysates (100 μg) and hypermethylated or non-methylated biotinylated probes (0.6 pmol) were incubated for 2 h at room temperature in the presence of streptavidin–agarose beads (Beaver Bio., China) to form DNA–protein–beads complexes; these were placed on a magnetic rack to promote the separation of the DNA–protein–beads complexes. The pulled-down complex was washed six times with 1 mL ice-cold tris-buffered saline and eluted in 80 μL protein elution buffer for 5 min at 95 °C. Proteins were separated by gel electrophoresis and identified by WB and mass spectrometry (MS) using LC–MS/MS (Eksigent ekspert nanoLC; AB Sciex TripleTOF 5600-plus) as described previously [25]. The MS data were analysed with Mascot software and mapped in the NCBInr database by Proteinpilot software.

### Electrophoretic mobility shift assay (EMSA)

Nucleoproteins from HCT116 cells were obtained using the nucleus protein extraction kit supplemented with the protease inhibitor phenylmethanesulfonyl fluoride (PMSF) at a final concentration of 1% (Beyotime, China). Protein quantification was determined using the bicinchoninic acid (BCA) method (Beyotime). A synthetic 5′-end biotin-labelled probe that was consistent with the TF-binding sequence was prepared. The 5′-end biotin-labelled probe, an unlabelled specific competitive probe, and the non-specific competitive mutation probe were generated by GeneCreate Biological Engineering Co (Wuhan, China) as previously described [24]. The sequences of all probes are shown in Additional file 1: Table S5. Briefly, in each 20 μL reaction, 20 fmol labelled probes were incubated with 2 μg nucleoprotein, together with 4 pmol specific competitive probe or non-specific mutation probe in an ionising environment, and 0.4–0.6 μg anti-CEBPB (Abcam, USA) and anti-TFCP2 (Proteintech, USA) antibodies. Migrating bands representing protein–DNA complexes were developed with a mixture of stable peroxide and luminol/enhancer solutions (LightShift Chemiluminescent EMSA kit, Thermo Fisher). An unlabelled probe at 50 × excess concentration was used as a specific competitor.

### Western blotting (WB) and Immunohistochemistry (IHC)

Total protein was extracted from cells and tissues using radioimmunoprecipitation (RIPA) lysis buffer (Beyotime, China) and quantitated using a BCA protein assay (Beyotime, China) as previously described [26]. Protein samples were denatured by boiling in a sodium dodecyl sulfate (SDS) sample buffer, separated by 12% SDS–polyacrylamide gel electrophoresis (PAGE), transferred to PVDF (polyvinylidene fluoride) membrane (Millipore, USA), and incubated with antibodies including anti-CPEB1 (Abcam, USA), anti-E-cadherin (Cell Signaling Technology (CST), USA), and anti-MMP-9 (CST, USA). GAPDH was used as the loading control. An IHC assay to detect Ki67 protein (Aoquan Med., China) was performed to evaluate cell proliferation in situ as previously described [27]. IHC staining was evaluated by two independent gastrointestinal pathologists. The staining intensity of each sample was scored as follows: 0, no staining; 1, weak staining; 2, moderate staining; and 3, strong staining. The percentage of positively stained tumour cells was also scored as follows: 1, < 10%; 2, 10% – 50%; 3, 51 – 75%; 4, > 75%. The IHC staining score was calculated as follows:

IHC staining score = IHC staining intensity score × score based on the number of positively stained tumour cells.

### Xenograft tumour mouse model

All animal experiments were reviewed and approved by the Institutional Animal Care and Use Committee (IACUC) of Affiliated Hospital of Nantong University. Four male nude BALB/c mice at 5 weeks of age (*n* = 4 per group; Nantong University Laboratory Animal Center, China) were anaesthetised via inhalation of a mixture of isoflurane/propylene glycol. HCT116 cells transfected with recombinant plasmid constructs containing pcDNA3.1-CPEB1 or pcDNA3.1 were injected subcutaneously into the back of each mouse (2.0 × 10^6^ cells in 200 μL PBS). The tumour sizes were assessed every 3 days by measuring in two dimensions; tumour volumes were estimated as the volume = (tumour length) × (tumour width)^2^/2. Mice were killed 36 days after implantation, and the tumours were collected and weighed. Slides were prepared and stained with hematoxylin and eosin (H&E). Levels of immunoreactive CPEB1, Ki67, MMP-9, and E-cadherin were determined by WB and IHC assays.

### Statistical analysis

Statistical assessments of differential DNA methylation and gene expression in CRC tumours and para-tumour tissue were performed using R (v3.6.3). All data from the functional experiments are shown as the mean ± standard deviation (SD). A value of *P* < 0.05 was considered statistically significant in two-tailed t-tests performed using SPSS 20.0. The false discovery rate (FDR) correction was used for multiple test corrections, as applicable. The bottoms, middles, and tops of the boxes in box-and-whisker plots represent the 25th (*Q*1), median (*Q*2), and 75th percentile (*Q*3), respectively.

The upper and the lower whiskers shown in the boxplots were calculated as follows:

$$\begin{gathered} {\text{upper}}\;{\text{whisker}}\, = \,\min (\max \left( x \right);Q3\, + \,1.5*{\text{IQR}}). \hfill \\ {\text{lower}}\;{\text{whisker}}\, = \,\max (\min \left( x \right);Q1{-}1.5*{\text{IQR}}), \hfill \\ \end{gathered}$$

where the interquartile range (IQR) = *Q*3–*Q*1.

## Discussion

**Fig. 7:**
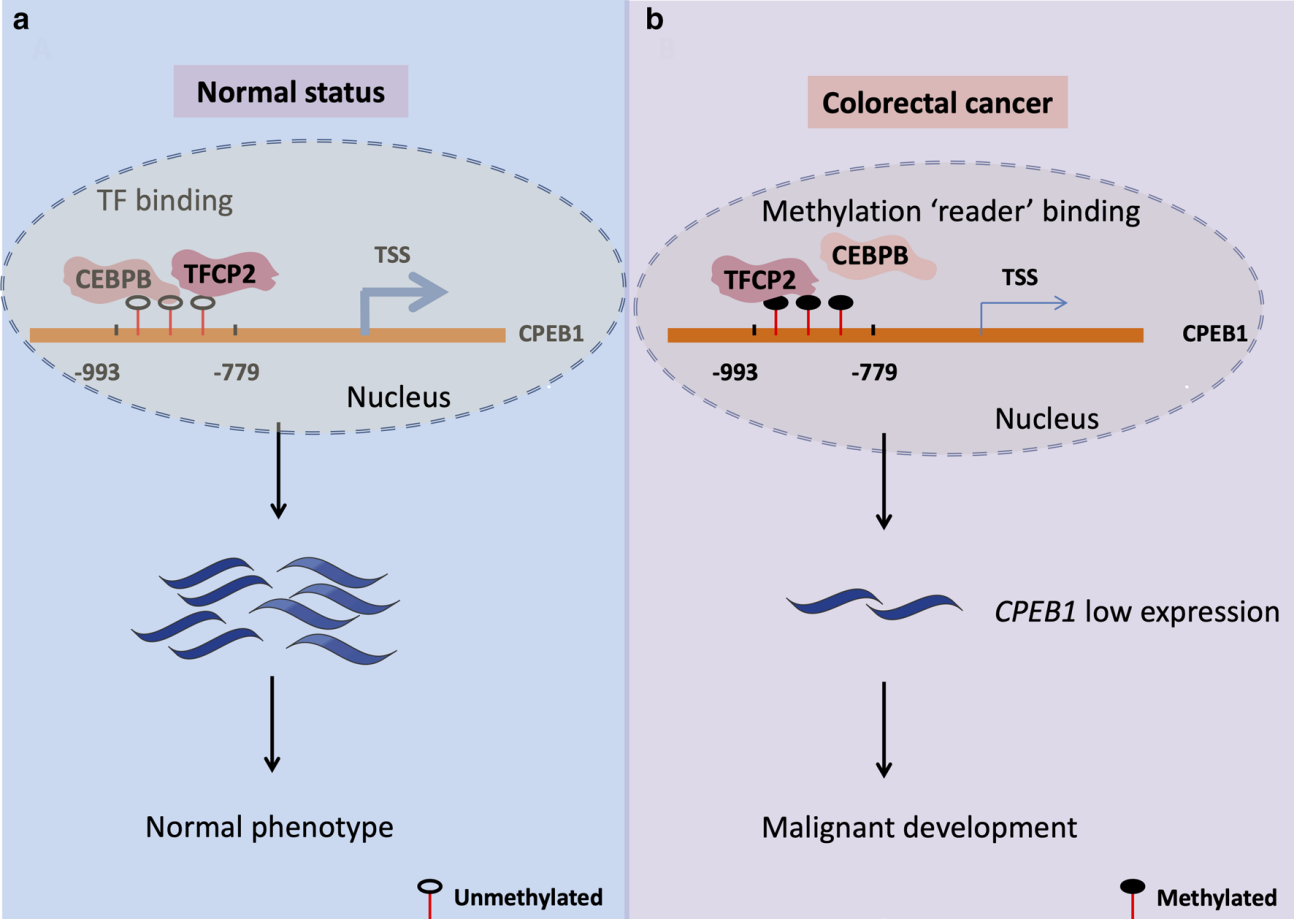
A model that explains the mechanism underlying *CPEB1* hypermethylation. The schematic diagram presented that epigenetic silencing of *CPEB1* enhances the capacity for malignant metastasis by reducing chromatin accessibility and thus CEBPB binding in colorectal cancer cells. **a** In healthy tissues, CEBPB binds to the *CPEB1* upstream region to initiate *CPEB1* transcription. **b** In CRC tissues, the core TF-binding region of CPEB1 is hypermethylated and thus inaccessible and unable to support CEBPB binding. However, the methylation reader TFCP2 can recognise and bind to the hypermethylated *CPEB1* core TF-binding region, thereby suppressing *CPEB1* expression and contributing to the potential for metastasis

Genome-wide alterations in DNA methylation have been widely reported in CRC in association with tumorigenesis and metastasis. However, the DNA methylation status of numerous TSGs and the contributions of hypermethylation to the pathogenesis of CRC remain largely unknown. Our study examined the hypermethylation status and expression of *CPEB1*, a putative TSG in CRC. The functional characterisation presented in this study confirmed the CRC tumour suppressor function of the *CPEB1* gene. We found that overexpression of *CPEB1* in CRC cell lines suppressed viability, colony formation, cellular invasion, and migration. *CPEB1* overexpression also resulted in the increased capacity for cell transformation, larger tumour volumes, and increased rates of tumour cell apoptosis in experiments performed both in vitro and in vivo. Furthermore, we found the TF, CEBPB could bind to the core promoter region of *CPEB1* and thereby regulate gene transcription. The hypermethylated form of the *CPEB1* promoter found in CRC cells did not support CEBPB binding, although this epigenetic modification facilitated interactions with TFCP2. These interactions resulted in diminished expression of *CPEB1* and enhanced CRC tumorigenesis and metastasis (Fig. 7).

Results from previous studies revealed that *CPEB1* played a major role in tumour suppression via its capacity to maintain *p53* mRNA polyadenylation and translation [16] and thus the functionality of the *p53* gene [28]. *CPEB1* has also been identified as a participant in the p53-mediated senescence of glioma cells [20]. Of note, *CPEB1* may also inhibit glioblastoma growth via its capacity to promote poly-A tail elongation and translation of the cell cycle inhibitor, *p27* [23]. *CPEB1* might also target the 3′-UTR of *SIRT1* to suppress the growth of hepatic carcinoma [22]. However, the functional significance of *CPEB1* in CRC has not been well characterised. Moreover, although previous studies have identified components of the *CPEB1* downstream signalling pathways involved in tumour suppression, the corresponding upstream regulatory events remain undefined. In this study, we used a multifaceted experimental approach to explore the molecular mechanisms employed by *CPEB1* as a TSG in CRC. Of note, we have demonstrated that *CPEB1* suppression was associated with CRC malignant transformation and that this role is dependent on hypermethylation of the TF-binding site in its promoter region. Collectively, our results suggest a role for demethylation drugs as a future therapeutic approach to CRC.

The TF, TFCP2, plays a crucial role in cancer incidence and development [29]. The *TFCP2*/*TFCP2L1*/*UBP1* TF subfamily is involved in various aspects of cancer development [30], including roles as pro-oncogenic factors in hepatocellular carcinoma as well as pancreatic and breast cancer. The actions of *TFCP2* may also promote cervical carcinogenesis and CRC [31]. *TFCP2* was identified as involved in the epithelial-mesenchymal transition and might also enhance angiogenesis [30]. Nonetheless, *TFCP2* can also act as a TSG, as exemplified by its capacity to inhibit melanoma growth [32]. These contrasting observations suggest that further research might be needed to further clarify the role of this TF subfamily in oncogenesis. In this study, our findings revealed that TFCP2 recognises and interacts with the hypermethylated *CPEB1* promoter and can suppress *CPEB1* transcription. Interestingly, TFCP2 binds to the methylated upstream region of *CPEB1*; this suggests that *TFCP2* might be a novel DNA methylation reader. However, the conclusion was based on only the in vitro studies only. Future in vivo experiments focused on the role of *TFCP2* in *CPEB1* regulation and CRC metastasis might provide important verification of this finding.

TFs could modulate the expression of target genes and play a key role in tumour development, differentiation, and metastasis [33, 34]. Methods that permit accurate detection of TF binding to specific target genes may have substantial potential for improved diagnosis and treatment of neoplastic disease [35, 36]. Our study predicted and confirmed that CEBPB binding to the -993 to -779 region of the *CPEB1* upstream region activated *CPEB1* transcription. Moreover, we demonstrated that hypermethylation of the *CPEB1* promoter region resulted in significantly diminished CEBPB binding of CEBPB to its DNA recognition elements, thereby leading to diminished expression of *CPEB1*. These results suggest that diminished capacity for CEBPB bindings may be an important mechanism that contributes to decreased *CPEB1* expression in CRC.

## Conclusions

In summary, our findings revealed hypermethylation within the upstream regions of *CPEB1* that was associated with its diminished expression in CRC tumours compared to para-tumour tissues. We identified CEBPB and TFCP2 as two critical TFs contributing to *CPEB1* regulation. The epigenetic modifications of *CPEB1* and subsequent transcriptional changes contribute significantly to CRC tumorigenesis and metastasis. Collectively, these results suggest that the development of *CPEB1* as a candidate biomarker and molecular target for CRC treatment is a subject worthy of further consideration.

## Supplementary Information

**Additional file 1**. Supplementary Results and Tables about this study.

**Additional file 2**. The excel of the methylation level for each sample in the validation dataset about this study.

## Data Availability

The data of the current study are available from the corresponding author upon reasonable request.
