# Supplementary material for: DNA hypermethylation contributes to colorectal cancer metastasis by regulating the binding of CEBPB and TFCP2 to the CPEB1 promoter
Source: Clin Epigenetics. 2021 Apr 23;13:89. doi: 10.1186/s13148-021-01071-z (PMC8063327; doi:10.1186/s13148-021-01071-z)
Supplement: Supplementary file 1 — Additional file 1. Supplementary Results and Tables about this study. [file 13148_2021_1071_MOESM1_ESM.docx]

**Supplementary files**


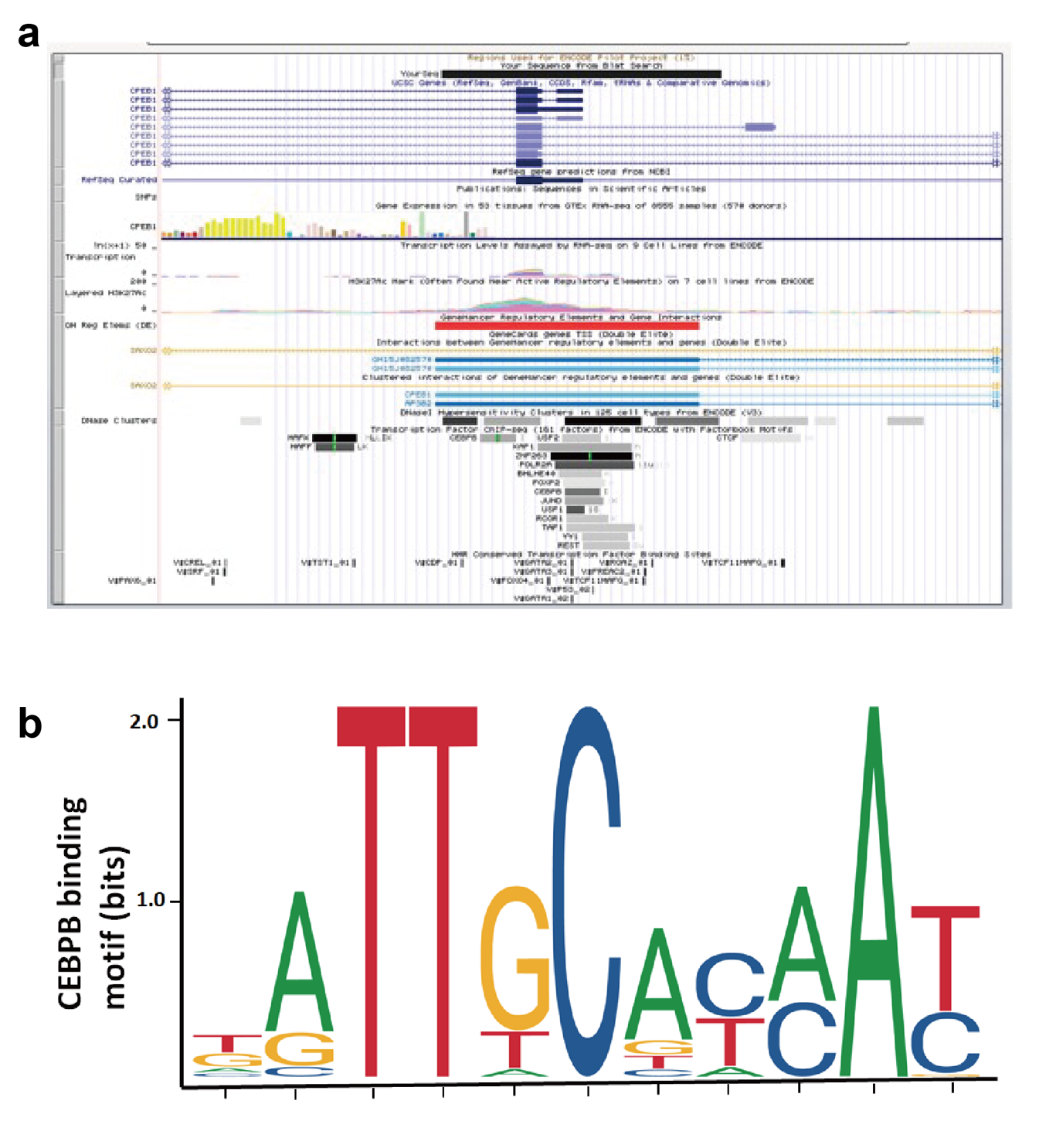


**Figure S1** **Predicted transcription factors (TFs) capable of binding to the *CPEB1* promoter and the predicted CEBPB binding motif**. **a)** TFs predicted to bind to the *CPEB1* promoter were retrieved from the ENCODE database of UCSC ([http://genome.ucsc.edu](http://genome.ucsc.edu/)). **b)** The CEBPB binding motif was predicted by JASPAR software.


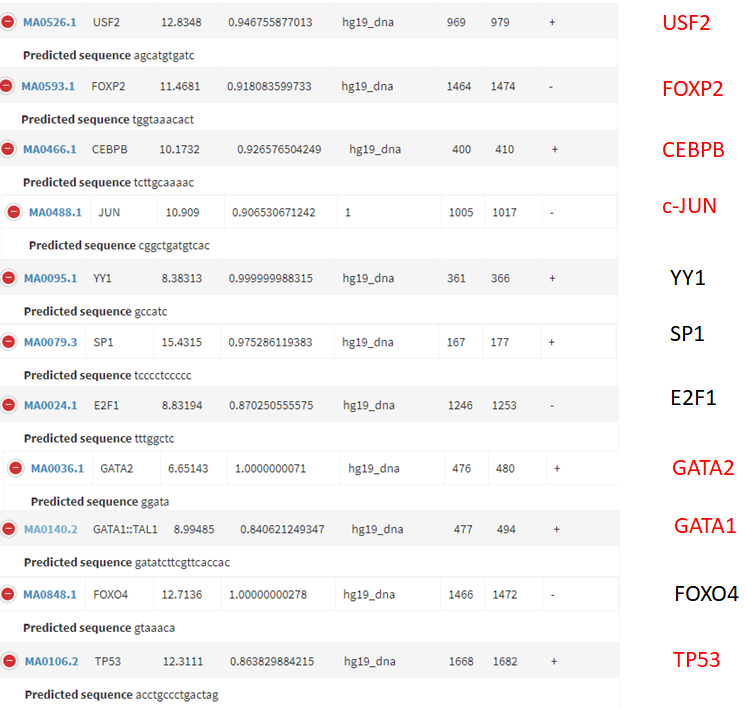


**Figure S2** **Predicted results of transcription factors (TFs) binding to the *CPEB1* promoter from the PROMO database.** Potential TFs capable of binding at the *CPEB1* promoter were retrieved from the PROMO database ([http://alggen.lsi.upc.es/cgi-bin/promo_v3/promo/](http://alggen.lsi.upc.es/cgi-bin/promo_v3/promo/promoinit.cgi?dirDB=TF_8.3)).


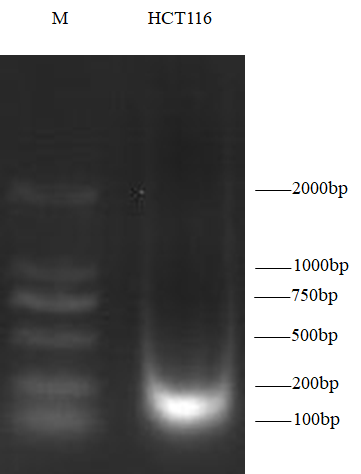


**Figure S3** **HCT116 cell DNA fragments after proteinase-K digestion in the ChIP experiment.** M, marker; HCT116, HCT116 cell line.


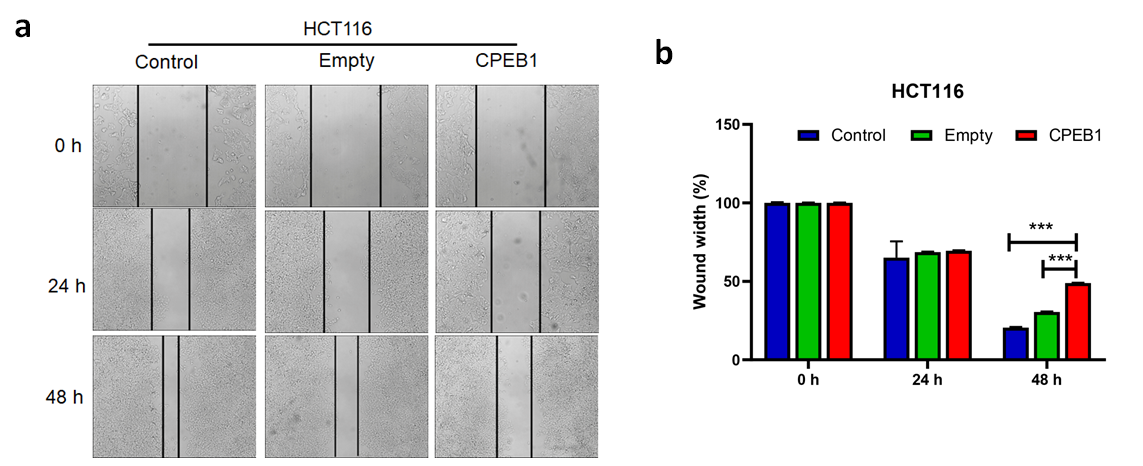


**Figure S4 Overexpression of *CPEB1* in HCT116 cells significantly inhibited wound healing.** Control, untransfected CRC cell line; Empty, CRC cells transfected with pcDNA3.1 (empty vector control); CPEB1, CRC cells transfected with pcDNA3.1-CPEB1; ****P* < 0.001.

**Supplementary Tables**

**Table S1 Primers used in the targeted bisulfite sequencing assay**

| Gene | Primer sequence (5'–3') |
| --- | --- |
| CPEB1-F | GGTYGGATGYGGAGTATTTGTG |
| CPEB1-R | AAAACTAAAACCCAAAACAAACAATAAC |
| Septin 9-F | GTTAGGGGGTTTAGGGGTTTTT |
| Septin 9-R | CAACAACCAACCCAACACC |

**Table S2 Primers utilized in the ChIP-PCR assay**

| Gene | Primer sequence (5'–3') |
| --- | --- |
| CPEB1-PCR1 F | TGCGTGAGGGCTGCTTGG |
| CPEB1-PCR1 R | GGACGACAGGGAGGAGACTTG |
| CPEB1-PCR2 F | GGCAAGTCTCCTCCCTGTCG |
| CPEB1-PCR2 R | GCAATAGAGAGCGGCATCATCC |
| CPEB1-PCR3 F | GTCGGCGGAGAGGACTTCAG |
| CPEB1-PCR3 R | CCAGGGAAAGACAGCACAACAG |

**Table S3 Primers to amplify the *CPEB1* promoter region in the DNA pull-down assay**

| Gene | Primer sequence (5'–3') |
| --- | --- |
| CPEB1-PR-F | TATCTCAGTGCATTATCCAGCCTC |
| CPEB1 PR-R | TAAGCATTGGTCTTGTGCAGC |

**Table S4 Sequences cloned into pGL3-basic vectors to identify the transcriptional activator of *CPEB1***

| TF | Type | Cloned sequence (5'–3') |
| --- | --- | --- |
| CEBPB | Wild-type | gtgcagactaacaactccagggaatttatcagggtaagtggaagagccatgcctgcctttacgtggagccatctgtcctgccacactcacagcttgcatttccatctcttgcaaaacagctccatgttgtatgtgtgtatggtggggagtgatgcaaaggggaacagaacaactgttgctgtggatatcttcgttcacca |
|  |  |  |
| CEBPB | Mutant | gtgcagactaacaactccagggaatttatcagggtaagtggaagagccatgcctgcctttacgtggagccatctgtcctgccacactcacagcttgcatttccatcctccatggggtagctccatgttgtatgtgtgtatggtggggagtgatgcaaaggggaacagaacaactgttgct gtggatatcttcgttcacca |
|  |  |  |
| GATA2 | Wild-type | gcttgcatttccatctcttgcaaaacagctccatgttgtatgtgtgtatggtggggagtgatgcaaaggggaacagaacaactgttgctgtggatatcttcgttcaccacccccatgcatccccttaccccagccaactcattctctggcactcacccgagtgtgtgctgctctgggctgag gaatctgagtcctgggtg |
|  |  |  |
| GATA2 | Mutant | gcttgcatttccatctcttgcaaaacagctccatgttgtatgtgtgtatggtggggagtgatgcaaaggggaacagaacaactgttgctgtaagcgtcttcgttcaccacccccatgcatccccttaccccagccaactcattctctggcactcacccgagtgtgtgctgctctgggctgag gaatctgagtcctgggtg |
|  |  |  |
| TP53 | Wild-type | cagagaaagggggtaagcgttggcagagtgggggagagtcactgggctgttccaggtacacctcaaaggacatggcccacaacccacatgccagagtgagacctgccctgactaggataatccctgtgcccaggttccctcatcccaacaagatgggtattagtcatgaacagataca gcggctcagctcctcccctccc |
|  |  |  |
| TP53 | Mutant | cagagaaagggggtaagcgttggcagagtgggggagagtcactgggctgttccaggtacacctcaaaggacatggcccacaacccacatgccagagtgaggttcatttcagtcgagataatccctgtgcccaggttccctcatcccaacaagatgggtattagtcatgaacagatacagc ggctcagctcctcccctccc |

**Base sequence highlighted with yellow colors indicated the potential binding motif to CEBPB, GATA2, and TP53, respectively.**

**Table S5 Primers used to amplify the probes in EMSA assay**

| Type | Primer sequence (5'–3') |
| --- | --- |
| Bio-Probe WT | TTGCTGGAGACCGGAGGGCGCTGC (Forward)  TAAGCATTGGTCTTGTGCAGC (Reverse) |
|  |  |
| Bio-Probe Mut | TTGCTGGTATGGCCTCCGCGCTGC (Forward)  TAAGCATTGGTCTTGTGCAGC (Reverse) |

**Table S6 The LS-MS/MS results of DNA pull-down assays in the *CPEB1* methylation group (236 TFs)**

| **Accession** | **Length** | **Mass** | **Unused** | **Coverage (%)** | **Peptide** | **Unique Peptide** |
| --- | --- | --- | --- | --- | --- | --- |
| sp\|Q8TAA3\|PSMA8_HUMAN | 256 | 28529.6 | 1.72 | 4.297 | 1 | 1 |
| sp\|Q13045\|FLII_HUMAN | 1269 | 144749.9 | 1.3 | 0.7092 | 1 | 1 |
| sp\|P12814\|ACTN1_HUMAN | 892 | 103056.7 | 2.05 | 8.52 | 7 | 1 |
| sp\|Q8NBF2\|NHLC2_HUMAN | 726 | 79442.9 | 1.89 | 1.928 | 1 | 1 |
| sp\|P36776\|LONM_HUMAN | 959 | 106488.4 | 2.06 | 1.251 | 1 | 1 |
| sp\|P54709\|AT1B3_HUMAN | 279 | 31512.3 | 1.48 | 3.584 | 1 | 1 |
| sp\|Q14684\|RRP1B_HUMAN | 758 | 84426.9 | 2.03 | 1.979 | 1 | 1 |
| sp\|Q01518\|CAP1_HUMAN | 475 | 51901.1 | 2.49 | 2.737 | 1 | 1 |
| sp\|P26358\|DNMT1_HUMAN | 1616 | 183163.6 | 1.41 | 0.495 | 1 | 1 |
| sp\|P08237\|PFKAM_HUMAN | 780 | 85181.9 | 2 | 2.692 | 2 | 1 |
| sp\|P40429\|RL13A_HUMAN | 203 | 23577.1 | 5.33 | 8.374 | 2 | 2 |
| sp\|Q9BW19\|KIFC1_HUMAN | 673 | 73747.1 | 2 | 2.08 | 1 | 1 |
| sp\|O95573\|ACSL3_HUMAN | 720 | 80419.4 | 3.6 | 4.167 | 2 | 2 |
| sp\|P51665\|PSMD7_HUMAN | 324 | 37025.2 | 2 | 4.012 | 1 | 1 |
| sp\|Q13867\|BLMH_HUMAN | 455 | 52561.9 | 2 | 2.418 | 1 | 1 |
| sp\|Q8IY81\|SPB1_HUMAN | 847 | 96557.7 | 2 | 0.9445 | 1 | 1 |
| sp\|Q9UKM9\|RALY_HUMAN | 306 | 32462.9 | 2 | 3.922 | 1 | 1 |
| sp\|P35221\|CTNA1_HUMAN | 906 | 100070.3 | 6.65 | 4.746 | 3 | 3 |
| sp\|P14866\|HNRPL_HUMAN | 589 | 64132.5 | 2.8 | 1.868 | 1 | 1 |
| sp\|P50914\|RL14_HUMAN | 215 | 23431.7 | 2 | 5.581 | 1 | 1 |
| sp\|P13798\|ACPH_HUMAN | 732 | 81223.9 | 2.04 | 1.639 | 1 | 1 |
| sp\|P13489\|RINI_HUMAN | 461 | 49972.7 | 2 | 2.386 | 1 | 1 |
| sp\|P46459\|NSF_HUMAN | 744 | 82593.6 | 8.44 | 7.796 | 5 | 5 |
| sp\|Q1KMD3\|HNRL2_HUMAN | 747 | 85104.2 | 3.31 | 3.748 | 3 | 3 |
| sp\|P18846\|ATF1_HUMAN | 271 | 29232.4 | 2.84 | 5.904 | 2 | 2 |
| sp\|Q5JTV8\|TOIP1_HUMAN | 583 | 66247.9 | 2 | 2.401 | 1 | 1 |
| sp\|Q16658\|FSCN1_HUMAN | 493 | 54529.5 | 2.79 | 5.071 | 2 | 2 |
| sp\|P35613\|BASI_HUMAN | 385 | 42200.1 | 2 | 3.636 | 1 | 1 |
| sp\|P62266\|RS23_HUMAN | 143 | 15807.5 | 3.83 | 15.38 | 2 | 2 |
| sp\|P42224\|STAT1_HUMAN | 750 | 87334.2 | 2.37 | 1.6 | 1 | 1 |
| sp\|Q9UL54\|TAOK2_HUMAN | 1235 | 138250.2 | 2 | 1.7 | 2 | 2 |
| sp\|P08559\|ODPA_HUMAN | 390 | 43295.3 | 2.29 | 5.641 | 2 | 2 |
| sp\|P49916\|DNLI3_HUMAN | 1009 | 112906 | 15.3 | 9.217 | 8 | 8 |
| sp\|P11216\|PYGB_HUMAN | 843 | 96695.2 | 2.05 | 3.796 | 3 | 1 |
| sp\|P36578\|RL4_HUMAN | 427 | 47696.9 | 2 | 2.81 | 1 | 1 |
| sp\|Q96P11\|NSUN5_HUMAN | 429 | 46691.2 | 2.47 | 3.263 | 1 | 1 |
| sp\|Q9NQ39\|RS10L_HUMAN | 176 | 20120.2 | 2 | 7.955 | 1 | 1 |
| sp\|O14744\|ANM5_HUMAN | 637 | 72683.2 | 2.01 | 2.041 | 1 | 1 |
| sp\|Q14165\|MLEC_HUMAN | 292 | 32233.6 | 1.81 | 4.11 | 1 | 1 |
| sp\|Q5SSJ5\|HP1B3_HUMAN | 553 | 61206.5 | 6.03 | 6.51 | 3 | 3 |
| sp\|P68366\|TBA4A_HUMAN | 448 | 49924 | 4.23 | 32.14 | 13 | 2 |
| sp\|P51659\|DHB4_HUMAN | 736 | 79685.7 | 1.91 | 1.223 | 1 | 1 |
| sp\|P49755\|TMEDA_HUMAN | 219 | 24975.7 | 2.04 | 4.566 | 1 | 1 |
| sp\|Q8WUM4\|PDC6I_HUMAN | 868 | 96022.3 | 5.04 | 2.189 | 2 | 2 |
| sp\|Q9Y6A4\|CFA20_HUMAN | 193 | 22774.2 | 2.01 | 6.218 | 1 | 1 |
| sp\|P51570\|GALK1_HUMAN | 392 | 42271.8 | 2 | 3.316 | 1 | 1 |
| sp\|Q9UBB9\|TFP11_HUMAN | 837 | 96819.2 | 5.15 | 3.584 | 3 | 3 |
| sp\|Q99873\|ANM1_HUMAN | 371 | 42461.3 | 4.81 | 10.24 | 3 | 3 |
| sp\|P50213\|IDH3A_HUMAN | 366 | 39591.4 | 2.05 | 3.279 | 1 | 1 |
| sp\|P54819\|KAD2_HUMAN | 239 | 26477.4 | 2 | 4.603 | 1 | 1 |
| sp\|Q16563\|SYPL1_HUMAN | 259 | 28565 | 1.82 | 4.247 | 1 | 1 |
| sp\|Q9Y6V7\|DDX49_HUMAN | 483 | 54225.8 | 2 | 2.277 | 1 | 1 |
| sp\|P42285\|MTREX_HUMAN | 1042 | 117803.8 | 2.37 | 1.344 | 1 | 1 |
| sp\|O00299\|CLIC1_HUMAN | 241 | 26922.5 | 6.35 | 18.67 | 4 | 4 |
| sp\|Q7L576\|CYFP1_HUMAN | 1253 | 145181.2 | 4.86 | 1.915 | 2 | 2 |
| sp\|P52292\|IMA1_HUMAN | 529 | 57861.4 | 2.02 | 3.403 | 1 | 1 |
| sp\|Q6UB35\|C1TM_HUMAN | 978 | 105789.1 | 4.28 | 2.556 | 2 | 2 |
| sp\|P08195\|4F2_HUMAN | 630 | 67993.3 | 4.21 | 4.603 | 3 | 3 |
| sp\|P21333\|FLNA_HUMAN | 2647 | 280737.6 | 2.13 | 0.4911 | 1 | 1 |
| sp\|P53618\|COPB_HUMAN | 953 | 107141.1 | 10.73 | 6.191 | 5 | 5 |
| sp\|P22087\|FBRL_HUMAN | 321 | 33784.1 | 4.95 | 9.969 | 3 | 3 |
| sp\|P51571\|SSRD_HUMAN | 173 | 18998.4 | 1.47 | 6.358 | 1 | 1 |
| sp\|O75489\|NDUS3_HUMAN | 264 | 30241.2 | 2 | 4.924 | 1 | 1 |
| sp\|Q9HAV0\|GBB4_HUMAN | 340 | 37566.8 | 1.64 | 3.235 | 1 | 1 |
| sp\|Q5T750\|XP32_HUMAN | 250 | 26237.9 | 1.55 | 3.2 | 1 | 1 |
| sp\|P60981\|DEST_HUMAN | 165 | 18505.5 | 2 | 6.667 | 1 | 1 |
| sp\|Q05639\|EF1A2_HUMAN | 463 | 50469.9 | 4.06 | 25.49 | 10 | 2 |
| sp\|Q9P015\|RM15_HUMAN | 296 | 33419.5 | 1.64 | 4.392 | 1 | 1 |
| sp\|P68104\|EF1A1_HUMAN | 462 | 50140.6 | 22.42 | 30.09 | 11 | 3 |
| sp\|Q9BQG0\|MBB1A_HUMAN | 1328 | 148853.2 | 1.7 | 0.8283 | 1 | 1 |
| sp\|P60900\|PSA6_HUMAN | 246 | 27399.2 | 2 | 5.285 | 1 | 1 |
| sp\|Q5JWF2\|GNAS1_HUMAN | 1037 | 111023.3 | 2.16 | 1.061 | 1 | 1 |
| sp\|P30041\|PRDX6_HUMAN | 224 | 25034.7 | 3.5 | 10.27 | 2 | 2 |
| sp\|Q15046\|SYK_HUMAN | 597 | 68047.5 | 2.37 | 2.68 | 2 | 2 |
| sp\|O75083\|WDR1_HUMAN | 606 | 66193.1 | 2.14 | 1.485 | 1 | 1 |
| sp\|Q6ZU15\|SEP14_HUMAN | 432 | 50024.6 | 2 | 2.083 | 1 | 1 |
| sp\|O60841\|IF2P_HUMAN | 1220 | 138825.9 | 2 | 0.9016 | 1 | 1 |
| sp\|P12277\|KCRB_HUMAN | 381 | 42643.9 | 2.78 | 2.887 | 1 | 1 |
| sp\|Q9H4A4\|AMPB_HUMAN | 650 | 72595.1 | 2 | 1.846 | 1 | 1 |
| sp\|Q8WTT2\|NOC3L_HUMAN | 800 | 92546.9 | 2.09 | 1.875 | 1 | 1 |
| sp\|Q99613\|EIF3C_HUMAN | 913 | 105342.9 | 8.06 | 5.038 | 4 | 4 |
| sp\|Q8N1F7\|NUP93_HUMAN | 819 | 93487.4 | 2.03 | 1.587 | 1 | 1 |
| sp\|P35249\|RFC4_HUMAN | 363 | 39681.3 | 1.89 | 3.03 | 1 | 1 |
| sp\|Q99798\|ACON_HUMAN | 780 | 85424.7 | 2.02 | 1.923 | 1 | 1 |
| sp\|O95757\|HS74L_HUMAN | 839 | 94511.7 | 2 | 4.887 | 3 | 1 |
| sp\|Q9Y5X1\|SNX9_HUMAN | 595 | 66591.2 | 1.7 | 2.185 | 1 | 1 |
| sp\|P62857\|RS28_HUMAN | 69 | 7841 | 1.8 | 17.39 | 1 | 1 |
| sp\|Q14764\|MVP_HUMAN | 893 | 99326.2 | 2.03 | 1.232 | 1 | 1 |
| sp\|P16615\|AT2A2_HUMAN | 1042 | 114755.8 | 3.68 | 2.687 | 2 | 2 |
| sp\|Q6P2E9\|EDC4_HUMAN | 1401 | 151659.7 | 2.77 | 0.9279 | 1 | 1 |
| sp\|Q96CT7\|CC124_HUMAN | 223 | 25835 | 2.73 | 5.83 | 1 | 1 |
| sp\|P35222\|CTNB1_HUMAN | 781 | 85495.9 | 2.11 | 6.146 | 5 | 2 |
| sp\|Q12800\|TFCP2_HUMAN | 502 | 57255.3 | 1.45 | 2.39 | 1 | 1 |
| sp\|P62753\|RS6_HUMAN | 249 | 28680.4 | 7.83 | 15.66 | 4 | 4 |
| sp\|P17858\|PFKAL_HUMAN | 780 | 85017.8 | 4.01 | 6.667 | 4 | 2 |
| sp\|Q13123\|RED_HUMAN | 557 | 65601.7 | 2.07 | 1.616 | 1 | 1 |
| sp\|Q16778\|H2B2E_HUMAN | 126 | 13920.1 | 10.45 | 23.81 | 5 | 2 |
| sp\|P61158\|ARP3_HUMAN | 418 | 47370.8 | 1.67 | 2.871 | 1 | 1 |
| sp\|Q9Y262\|EIF3L_HUMAN | 564 | 66726.5 | 7.05 | 7.092 | 4 | 4 |
| sp\|Q9UQM7\|KCC2A_HUMAN | 478 | 54087.3 | 1.89 | 3.975 | 2 | 1 |
| sp\|P08758\|ANXA5_HUMAN | 320 | 35936.4 | 7.67 | 13.12 | 4 | 4 |
| sp\|P15559\|NQO1_HUMAN | 274 | 30867.4 | 3.07 | 6.934 | 2 | 2 |
| sp\|P55084\|ECHB_HUMAN | 474 | 51294 | 4 | 4.641 | 2 | 2 |
| sp\|P61964\|WDR5_HUMAN | 334 | 36588.1 | 1.41 | 6.886 | 2 | 2 |
| sp\|Q9NRH3\|TBG2_HUMAN | 451 | 51091.3 | 1.55 | 1.996 | 1 | 1 |
| sp\|P60953\|CDC42_HUMAN | 191 | 21258.4 | 2 | 5.236 | 1 | 1 |
| sp\|P47756\|CAPZB_HUMAN | 277 | 31350.2 | 1.3 | 3.61 | 1 | 1 |
| sp\|Q9H9B4\|SFXN1_HUMAN | 322 | 35619.1 | 2.62 | 4.037 | 1 | 1 |
| sp\|O00410\|IPO5_HUMAN | 1097 | 123628.9 | 3.13 | 1.732 | 2 | 2 |
| sp\|O75390\|CISY_HUMAN | 466 | 51712 | 5.29 | 6.867 | 3 | 3 |
| sp\|O43395\|PRPF3_HUMAN | 683 | 77528.4 | 1.6 | 1.025 | 1 | 1 |
| sp\|P43304\|GPDM_HUMAN | 727 | 80852 | 1.77 | 3.439 | 2 | 2 |
| sp\|P36542\|ATPG_HUMAN | 298 | 32995.7 | 2 | 4.027 | 1 | 1 |
| sp\|Q04837\|SSBP_HUMAN | 148 | 17259.6 | 2 | 10.14 | 1 | 1 |
| sp\|P18887\|XRCC1_HUMAN | 633 | 69475.9 | 2.61 | 1.58 | 1 | 1 |
| sp\|Q9Y6E2\|BZW2_HUMAN | 419 | 48162 | 5.12 | 5.251 | 3 | 3 |
| sp\|P17174\|AATC_HUMAN | 413 | 46247.1 | 1.77 | 4.116 | 2 | 2 |
| sp\|P19105\|ML12A_HUMAN | 171 | 19794 | 2 | 5.848 | 1 | 1 |
| sp\|Q9NQC3\|RTN4_HUMAN | 1192 | 129929.8 | 2 | 1.091 | 1 | 1 |
| sp\|Q9NQW7\|XPP1_HUMAN | 623 | 69917.2 | 1.62 | 1.766 | 1 | 1 |
| sp\|Q9UKN8\|TF3C4_HUMAN | 822 | 91981.6 | 1.92 | 1.095 | 1 | 1 |
| sp\|Q15269\|PWP2_HUMAN | 919 | 102451.2 | 2.33 | 1.088 | 1 | 1 |
| sp\|P04181\|OAT_HUMAN | 439 | 48534.4 | 2.32 | 3.189 | 1 | 1 |
| sp\|Q9HDC9\|APMAP_HUMAN | 416 | 46479.9 | 2 | 2.885 | 1 | 1 |
| sp\|Q16656\|NRF1_HUMAN | 503 | 53540.7 | 2 | 2.386 | 1 | 1 |
| sp\|Q9UNM6\|PSD13_HUMAN | 376 | 42945.2 | 2.5 | 2.66 | 1 | 1 |
| sp\|P62851\|RS25_HUMAN | 125 | 13742 | 4.7 | 16 | 2 | 2 |
| sp\|Q15149\|PLEC_HUMAN | 4684 | 531785.9 | 3.22 | 0.3416 | 1 | 1 |
| sp\|Q9NYL9\|TMOD3_HUMAN | 352 | 39594.4 | 2 | 3.125 | 1 | 1 |
| sp\|Q9Y6M1\|IF2B2_HUMAN | 599 | 66121 | 2.17 | 2.17 | 1 | 1 |
| sp\|O00139\|KIF2A_HUMAN | 706 | 79953.9 | 2.03 | 1.558 | 1 | 1 |
| sp\|Q00059\|TFAM_HUMAN | 246 | 29096.4 | 1.71 | 2.846 | 1 | 1 |
| sp\|Q9Y277\|VDAC3_HUMAN | 283 | 30658.5 | 4.51 | 8.481 | 2 | 2 |
| sp\|Q9UHR5\|S30BP_HUMAN | 308 | 33870.1 | 2.04 | 4.87 | 1 | 1 |
| sp\|P16435\|NCPR_HUMAN | 677 | 76689.1 | 2 | 1.329 | 1 | 1 |
| sp\|Q9NVI7\|ATD3A_HUMAN | 634 | 71368.6 | 11.09 | 8.36 | 5 | 5 |
| sp\|P36957\|ODO2_HUMAN | 453 | 48754.9 | 1.6 | 1.766 | 1 | 1 |
| sp\|O00303\|EIF3F_HUMAN | 357 | 37563.5 | 1.43 | 4.762 | 1 | 1 |
| sp\|P21127\|CD11B_HUMAN | 795 | 92619.2 | 2.51 | 1.635 | 1 | 1 |
| sp\|Q8IUX4\|ABC3F_HUMAN | 373 | 45019.8 | 2 | 3.217 | 1 | 1 |
| sp\|P61353\|RL27_HUMAN | 136 | 15797.6 | 4 | 12.5 | 2 | 2 |
| sp\|P62910\|RL32_HUMAN | 135 | 15859.7 | 1.47 | 5.185 | 1 | 1 |
| sp\|P28838\|AMPL_HUMAN | 519 | 56165.8 | 3.17 | 5.202 | 2 | 2 |
| sp\|P62263\|RS14_HUMAN | 151 | 16272.6 | 1.85 | 7.285 | 1 | 1 |
| sp\|P47897\|SYQ_HUMAN | 775 | 87798 | 1.78 | 1.29 | 1 | 1 |
| sp\|Q92979\|NEP1_HUMAN | 244 | 26719.9 | 3.27 | 10.25 | 2 | 2 |
| sp\|P46013\|KI67_HUMAN | 3256 | 358691 | 2.73 | 0.6757 | 1 | 1 |
| sp\|Q9UN37\|VPS4A_HUMAN | 437 | 48897.5 | 2.03 | 2.746 | 1 | 1 |
| sp\|P56192\|SYMC_HUMAN | 900 | 101114.9 | 2 | 1.333 | 1 | 1 |
| sp\|Q09028\|RBBP4_HUMAN | 425 | 47655.3 | 6.01 | 8.235 | 3 | 3 |
| sp\|P50570\|DYN2_HUMAN | 870 | 98063.3 | 3.1 | 2.414 | 2 | 2 |
| sp\|P35998\|PRS7_HUMAN | 433 | 48633.4 | 2 | 2.54 | 1 | 1 |
| sp\|P19367\|HXK1_HUMAN | 917 | 102485.1 | 1.33 | 0.8724 | 1 | 1 |
| sp\|Q96SB4\|SRPK1_HUMAN | 655 | 74324.3 | 2.03 | 2.137 | 1 | 1 |
| sp\|O00567\|NOP56_HUMAN | 594 | 66049.3 | 2.57 | 2.189 | 1 | 1 |
| sp\|P62304\|RUXE_HUMAN | 92 | 10803.6 | 2 | 11.96 | 1 | 1 |
| sp\|P11413\|G6PD_HUMAN | 515 | 59256.3 | 2.25 | 1.553 | 1 | 1 |
| sp\|P14174\|MIF_HUMAN | 115 | 12476.2 | 2 | 7.826 | 1 | 1 |
| sp\|P09960\|LKHA4_HUMAN | 611 | 69284.6 | 3.08 | 3.764 | 2 | 2 |
| sp\|P19525\|E2AK2_HUMAN | 551 | 62093.7 | 2 | 2.359 | 1 | 1 |
| sp\|O00231\|PSD11_HUMAN | 422 | 47463.2 | 2.4 | 2.844 | 1 | 1 |
| sp\|Q9NSE4\|SYIM_HUMAN | 1012 | 113790.6 | 2.28 | 1.087 | 1 | 1 |
| sp\|P62191\|PRS4_HUMAN | 440 | 49184.1 | 4.03 | 5.455 | 2 | 2 |
| sp\|P43307\|SSRA_HUMAN | 286 | 32235.1 | 1.68 | 2.797 | 1 | 1 |
| sp\|Q9UNX4\|WDR3_HUMAN | 943 | 106097.9 | 3.36 | 2.757 | 2 | 2 |
| sp\|Q9UKK9\|NUDT5_HUMAN | 219 | 24327.4 | 2.02 | 6.849 | 1 | 1 |
| sp\|Q93077\|H2A1C_HUMAN | 130 | 14105.4 | 2 | 20.77 | 3 | 1 |
| sp\|P61604\|CH10_HUMAN | 102 | 10931.6 | 2 | 13.73 | 1 | 1 |
| sp\|O00571\|DDX3X_HUMAN | 662 | 73242.8 | 7.95 | 8.157 | 4 | 4 |
| sp\|P62829\|RL23_HUMAN | 140 | 14865.3 | 2.42 | 12.86 | 2 | 2 |
| sp\|Q16555\|DPYL2_HUMAN | 572 | 62293.1 | 1.54 | 2.797 | 1 | 1 |
| sp\|O43747\|AP1G1_HUMAN | 822 | 91350.5 | 2.92 | 2.19 | 2 | 2 |
| sp\|P12235\|ADT1_HUMAN | 298 | 33064.3 | 1.44 | 10.4 | 3 | 1 |
| sp\|Q12788\|TBL3_HUMAN | 808 | 89033.9 | 6.02 | 5.198 | 3 | 3 |
| sp\|Q6NXG1\|ESRP1_HUMAN | 681 | 75584.7 | 2.16 | 2.203 | 1 | 1 |
| sp\|Q9Y446\|PKP3_HUMAN | 797 | 87081.1 | 4 | 3.137 | 2 | 2 |
| sp\|P16188\|1A30_HUMAN | 365 | 40904.5 | 2.01 | 3.562 | 1 | 1 |
| sp\|P32119\|PRDX2_HUMAN | 198 | 21891.7 | 2.29 | 9.596 | 2 | 1 |
| sp\|Q8N1G4\|LRC47_HUMAN | 583 | 63472.2 | 2.1 | 2.058 | 1 | 1 |
| sp\|P04066\|FUCO_HUMAN | 466 | 53688.5 | 1.44 | 2.361 | 1 | 1 |
| sp\|P02786\|TFR1_HUMAN | 760 | 84870.7 | 3.85 | 2.895 | 2 | 2 |
| sp\|O76021\|RL1D1_HUMAN | 490 | 54972 | 4.82 | 6.531 | 2 | 2 |
| sp\|O43242\|PSMD3_HUMAN | 534 | 60977 | 2.48 | 2.434 | 1 | 1 |
| sp\|P14735\|IDE_HUMAN | 1019 | 117967.5 | 1.72 | 1.178 | 1 | 1 |
| sp\|O43252\|PAPS1_HUMAN | 624 | 70832.7 | 3.2 | 4.006 | 2 | 2 |
| sp\|Q13509\|TBB3_HUMAN | 450 | 50432.4 | 2 | 22.89 | 9 | 1 |
| sp\|Q15084\|PDIA6_HUMAN | 440 | 48120.9 | 4 | 6.364 | 2 | 2 |
| sp\|P62333\|PRS10_HUMAN | 389 | 44172.6 | 2.43 | 2.828 | 1 | 1 |
| sp\|P49748\|ACADV_HUMAN | 655 | 70389.6 | 11.57 | 10.84 | 6 | 6 |
| sp\|P26006\|ITA3_HUMAN | 1051 | 116611.3 | 1.59 | 0.8563 | 1 | 1 |
| sp\|P21399\|ACOC_HUMAN | 889 | 98398.1 | 4.03 | 3.712 | 2 | 2 |
| sp\|P07954\|FUMH_HUMAN | 510 | 54636.6 | 2.06 | 3.529 | 1 | 1 |
| sp\|Q07020\|RL18_HUMAN | 188 | 21634.3 | 8.71 | 25 | 4 | 4 |
| sp\|Q92616\|GCN1_HUMAN | 2671 | 292755 | 5.19 | 0.8985 | 2 | 2 |
| sp\|P12004\|PCNA_HUMAN | 261 | 28768.5 | 9.19 | 20.69 | 5 | 5 |
| sp\|P31350\|RIR2_HUMAN | 389 | 44877.2 | 2 | 3.342 | 1 | 1 |
| sp\|P06746\|DPOLB_HUMAN | 335 | 38177.3 | 2.57 | 6.866 | 2 | 2 |
| sp\|Q9H3U1\|UN45A_HUMAN | 944 | 103075.9 | 3.89 | 3.072 | 2 | 2 |
| sp\|P53396\|ACLY_HUMAN | 1101 | 120838.3 | 3.64 | 2.361 | 3 | 3 |
| sp\|Q9Y4W6\|AFG32_HUMAN | 797 | 88583 | 2.86 | 2.384 | 2 | 2 |
| sp\|P78527\|PRKDC_HUMAN | 4128 | 469084.2 | 1.62 | 0.2665 | 1 | 1 |
| sp\|P05198\|IF2A_HUMAN | 315 | 36111.8 | 3.89 | 6.349 | 2 | 2 |
| sp\|Q12905\|ILF2_HUMAN | 390 | 43061.8 | 6.98 | 13.59 | 4 | 4 |
| sp\|P46778\|RL21_HUMAN | 160 | 18564.8 | 4.34 | 16.25 | 2 | 2 |
| sp\|O00170\|AIP_HUMAN | 330 | 37635.7 | 2 | 4.242 | 1 | 1 |
| sp\|Q14137\|BOP1_HUMAN | 746 | 83628.8 | 1.43 | 1.877 | 1 | 1 |
| sp\|Q9UQ80\|PA2G4_HUMAN | 394 | 43786.6 | 1.6 | 2.792 | 1 | 1 |
| sp\|P31689\|DNJA1_HUMAN | 397 | 44868 | 2.03 | 3.275 | 1 | 1 |
| sp\|Q08945\|SSRP1_HUMAN | 709 | 81074.2 | 2.59 | 1.551 | 1 | 1 |
| sp\|Q08J23\|NSUN2_HUMAN | 767 | 86470 | 1.86 | 1.434 | 1 | 1 |
| sp\|O43172\|PRP4_HUMAN | 522 | 58448.7 | 5.28 | 6.897 | 3 | 3 |
| sp\|P61981\|1433G_HUMAN | 247 | 28302.3 | 4 | 9.312 | 2 | 2 |
| sp\|P53992\|SC24C_HUMAN | 1094 | 118323.8 | 2 | 1.28 | 1 | 1 |
| sp\|Q09161\|NCBP1_HUMAN | 790 | 91838.6 | 3.47 | 2.911 | 2 | 2 |
| sp\|P50395\|GDIB_HUMAN | 445 | 50662.8 | 4.36 | 4.494 | 2 | 2 |
| sp\|P63173\|RL38_HUMAN | 70 | 8217.8 | 2.43 | 17.14 | 1 | 1 |
| sp\|P83731\|RL24_HUMAN | 157 | 17778.8 | 2.37 | 8.28 | 1 | 1 |
| sp\|O75821\|EIF3G_HUMAN | 320 | 35610.7 | 1.96 | 2.812 | 1 | 1 |
| sp\|Q99879\|H2B1M_HUMAN | 126 | 13989.2 | 2.66 | 23.81 | 4 | 1 |
| sp\|Q9UK22\|FBX2_HUMAN | 296 | 33327.5 | 2 | 5.068 | 1 | 1 |
| sp\|O60506\|HNRPQ_HUMAN | 623 | 69602.3 | 15.48 | 12.84 | 8 | 4 |
| sp\|P35250\|RFC2_HUMAN | 354 | 39156.9 | 2 | 4.802 | 1 | 1 |
| sp\|Q13442\|HAP28_HUMAN | 181 | 20629.9 | 1.49 | 7.182 | 1 | 1 |
| sp\|Q92973\|TNPO1_HUMAN | 898 | 102354 | 2.38 | 1.336 | 1 | 1 |
| sp\|Q09666\|AHNK_HUMAN | 5890 | 629098.1 | 2.44 | 0.1868 | 1 | 1 |
| sp\|P08133\|ANXA6_HUMAN | 673 | 75872.5 | 3.66 | 4.755 | 3 | 3 |
| sp\|Q14847\|LASP1_HUMAN | 261 | 29717.1 | 2 | 4.981 | 1 | 1 |
| sp\|Q9Y570\|PPME1_HUMAN | 386 | 42315.1 | 2.02 | 3.368 | 1 | 1 |
| sp\|P46087\|NOP2_HUMAN | 812 | 89301.1 | 2.02 | 1.108 | 1 | 1 |
| sp\|P22695\|QCR2_HUMAN | 453 | 48442.6 | 1.86 | 2.428 | 1 | 1 |
| sp\|P31930\|QCR1_HUMAN | 480 | 52645.3 | 4 | 4.167 | 2 | 2 |
| sp\|Q13263\|TIF1B_HUMAN | 835 | 88548.8 | 3.14 | 2.994 | 2 | 2 |
| sp\|P51553\|IDH3G_HUMAN | 393 | 42794 | 1.92 | 2.799 | 1 | 1 |
| sp\|Q13308\|PTK7_HUMAN | 1070 | 118390.7 | 1.77 | 1.121 | 1 | 1 |
| sp\|Q9BVP2\|GNL3_HUMAN | 549 | 61992.7 | 3.51 | 3.461 | 2 | 2 |
| sp\|P13674\|P4HA1_HUMAN | 534 | 61048.8 | 7.8 | 9.925 | 4 | 4 |
